# GART expression is associated with a proliferative and purine-metabolic ISC-like epithelial state in ulcerative colitis

**DOI:** 10.3389/fimmu.2026.1913904

**Published:** 2026-10-05

**Authors:** Jinchen Chong, Haoyu Ding, Jiaze Ma, Yuan Li, Jie Pan, Yang Li, Yugen Chen

**Affiliations:** 1 The Affiliated Hospital of Nanjing University of Chinese Medicine, Nanjing, China; 2 No. 1 Clinical Medical College, Nanjing University of Chinese Medicine, Nanjing, China; 3 Department of Colorectal Surgery, The Affiliated Hospital of Nanjing University of Chinese Medicine, Jiangsu Province Hospital of Chinese Medicine, Nanjing, China; 4 Endoscopy Center, The Affiliated Hospital of Nanjing University of Chinese Medicine (Jiangsu Province Hospital of Chinese Medicine), Nanjing, China; 5 Jiangsu Province Key Laboratory of Tumor Systems Biology and Chinese Medicine, The Affiliated Hospital of Nanjing University of Chinese Medicine, Nanjing, China; 6 Jiangsu Collaborative Innovation Center of Chinese Medicine in Prevention and Treatment of Tumor, The Affiliated Hospital of Nanjing University of Chinese Medicine, Nanjing, China

**Keywords:** epithelial proliferation, GART, intestinal stem cell-like epithelial cells, purine metabolism, ulcerative colitis

## Abstract

**Objective:**

To characterize the epithelial state associated with GART expression in ulcerative colitis (UC) and determine whether this association reflects a GART-specific program or a broader proliferative and purine-metabolic phenotype.

**Methods:**

Four independent bulk transcriptomic cohorts were analyzed and synthesized by random-effects meta-analysis. Human colonic scRNA-seq datasets (GSE214695 and SCP259) were integrated, with principal comparisons repeated using donor-aware pseudobulk or mixed-effects approaches and adjustment for sequencing depth, gene detection, and cell-cycle covariates. GART detection was defined from raw counts and tested using alternative thresholds. Pseudotime, scTenifoldKnk, CellChat/LIANA, and transcriptional-network analyses were used as exploratory analyses. Spatial transcriptomics (GSE189184), human colonic multiplex immunofluorescence, and acute DSS-induced colitis tissues were used for tissue-level localization.

**Results:**

Across the four bulk cohorts, GART expression showed an overall increase in UC (pooled Hedges’ g = 0.743, 95% CI 0.239–1.248; P = 0.0183). In the single-cell atlas, GART detection localized preferentially to an ISC-like epithelial compartment, although the magnitude of the UC-associated detection difference varied between cohorts. Within UC ISC-like cells, donor-paired pseudobulk analyses identified proliferative and nucleotide-metabolic transcriptional differences associated with GART detection. Across the broader ISC-like epithelial atlas, donor-aware module analyses showed positive associations of GART detection with purine-biosynthesis, DNA-replication, DNA-repair, and mitotic programs, and these associations remained positive within cell-cycle strata. Comparison with other *de novo* purine-synthesis enzymes did not support a uniquely GART-specific alteration. Exploratory network analyses linked the GART-associated state to proliferative and nucleotide-metabolic programs but did not establish functional dependence. In seven independent spatial donors, the single-cell-derived epithelial program was consistently higher in GART-detected than GART-undetected epithelial regions (mean within-donor difference = 0.0262, 95% CI 0.0140–0.0407; exact P = 0.0156). Human tissue immunofluorescence further showed co-enrichment of GART with OLFM4-positive ISC-like, MYBL2/Ki67-positive proliferative, and MIF/CD74-associated inflammatory regions across the corresponding panel-specific participant sets.

**Conclusion:**

GART identifies a proliferative and purine-metabolic ISC-like epithelial state in UC but is not established as a specific driver of epithelial regeneration. The findings support an association between GART detection, nucleotide-demand programs, proliferative epithelial activity, and the inflammatory tissue context, providing a framework for future direct functional studies.

## Introduction

Ulcerative colitis (UC) is a chronic inflammatory bowel disease characterized by recurrent inflammation of the colorectal mucosa, epithelial injury, and impaired barrier integrity (1). The global burden of UC has increased in recent decades, with environmental exposures, lifestyle factors, and alterations in the gut microbiome contributing to disease risk (2). Current treatment options include 5-aminosalicylic acid, corticosteroids, immunosuppressants, biologics, and small-molecule agents (3). Although these therapies can effectively control inflammation and induce remission in many patients, a substantial proportion experience incomplete response or recurrent disease. Therefore, defining the epithelial states that accompany inflammatory mucosal injury and their relationship to restoration of tissue integrity may provide further insight into UC pathobiology.

The intestinal epithelial barrier is central to maintaining intestinal homeostasis (4). During UC, persistent inflammation disrupts crypt architecture, epithelial differentiation, and barrier function, thereby contributing to continued mucosal injury. Crypt-based stem and progenitor cells, including ISC-like epithelial populations, play important roles in epithelial turnover and the response to tissue injury (5–7). Following injury, epithelial cells can adopt injury-associated proliferative or transitional states characterized by altered cell-cycle activity, DNA replication, nucleotide demand, and metabolic remodeling. Such changes may accompany compensatory epithelial responses to mucosal damage. How these injury-associated epithelial states are metabolically organized in UC, and how they relate to the local inflammatory microenvironment, remains incompletely understood.

GART is a multifunctional enzyme in the *de novo* purine biosynthesis pathway and participates in reactions required for nucleotide synthesis and one-carbon utilization (8). Because DNA replication and cell-cycle progression increase nucleotide demand, *de novo* purine biosynthesis is closely linked to proliferative cellular activity. However, the expression pattern and cellular context of GART in the UC epithelium remain poorly characterized. In particular, little is known about how GART is distributed across epithelial cell states or which proliferative and metabolic features accompany its expression during inflammatory mucosal injury. Defining this cellular and molecular context may provide insight into how *de novo* purine metabolism is organized within epithelial responses to UC.

In this study, we integrated four independent bulk transcriptomic cohorts with two human colonic single-cell RNA-seq datasets (GSE214695 and SCP259) to characterize the epithelial distribution and molecular context of GART in UC. We focused on ISC-like epithelial cells and examined the transcriptional programs associated with GART detection using donor-aware analyses that accounted for technical and cell-cycle-related variation. Parallel analyses of other enzymes in the *de novo* purine biosynthesis pathway were used to place the GART-associated findings in a broader pathway context. Pseudotime, in silico perturbation, cell–cell communication, and transcriptional-network analyses were used as exploratory approaches to further characterize the associated epithelial state. Finally, spatial transcriptomics, human colonic multiplex immunofluorescence, and DSS-induced colitis tissues were used to examine the tissue-level localization of GART and its relationship with ISC-like, proliferative, purine-metabolic, and inflammatory features. Together, this multimodal framework was used to characterize the GART-associated epithelial state across transcriptomic and tissue contexts.

## Methods

### Ethics statement

The human tissue study and animal experiments were approved by the Ethics Committee of Jiangsu Province Hospital of Chinese Medicine. The animal study was conducted under approval no. 2025DW-034-01 (approved March 24, 2025), and the human tissue study under approval no. 2022NL-083-01 (approved May 25, 2022). Written informed consent was obtained from all participants before tissue collection, and all human data were de-identified before analysis.

### Analysis of bulk transcriptome data

Publicly available human intestinal mucosal bulk transcriptomic datasets were obtained from the Gene Expression Omnibus (GEO), including GSE24287, GSE36807, GSE37283, and GSE87466 (9–12). For GSE37283, samples with UC-associated neoplasia were excluded, and only non-neoplastic UC and healthy control samples were retained. The exact samples included in each cohort, together with available information on disease activity, biopsy location, treatment exposure, and exclusion criteria, are summarized in **Supplementary Table S1**.

Microarray data were retrieved through the documented MINiML workflow. Log2 transformation and quantile normalization with preprocessCore::normalize.quantiles were applied conditionally according to the distributed scale and preprocessing state. Probe identifiers were mapped to gene symbols using the corresponding platform annotation; probes mapping to multiple genes were excluded, and multiple retained probes mapping uniquely to the same gene were summarized by their arithmetic mean. Technical batch effects were corrected with limma::removeBatchEffect when applicable (historical workflow: R 4.0.3, preprocessCore 1.50.0, and limma 3.44.3). GSE24287 used GEO source-level print-tip-loess processed log-scale values, whereas GSE36807 and GSE37283 used RMA-processed values. The corresponding GART probe identifiers, source processing, mapping rules, and aggregation procedures for each cohort are provided in **Supplementary Table S1** and the public repository. For each cohort, the difference in GART expression between UC and healthy control samples was summarized as Hedges’ g with a 95% confidence interval. Cohort-specific effect sizes were synthesized using a random-effects meta-analysis with restricted maximum likelihood estimation of between-study variance and Hartung–Knapp adjustment for inference. Statistical heterogeneity was assessed using Cochran’s Q, I^2^, and τ^2^. All analyses were two-sided.

### Integration and preprocessing of scRNA-seq data

Publicly available human colonic scRNA-seq datasets GSE214695 and SCP259 were integrated for downstream analysis (13–15). Dataset-specific quality-control procedures followed the preprocessing workflows of the respective source studies. For GSE214695, Cell Ranger-filtered matrices were generated by the source workflow, which applied per-sample scDblFinder (v1.8.0, default parameters) and retained cells with >100 detected genes and <65% mitochondrial transcripts; no additional hard UMI-count threshold was applied. For SCP259, we used the processed release generated by the original study, in which cells with fewer than 250 detected genes were excluded, mixed-lineage doublet clusters were iteratively identified and removed, and putative ambient-RNA contaminants were filtered using the source study’s contamination model.

After dataset-specific source preprocessing and subsequent cross-dataset integration, expression data were normalized using LogNormalize with a scale factor of 10,000, 2,000 highly variable genes were selected, and 30 principal components were retained. Harmony dimensions 1–30 were used for the final low-dimensional representation (16). The verified software environment comprised R 4.4.2, Seurat 5.4.0, SeuratObject 5.3.0, and harmony 1.2.4.

The final atlas analyzed here comprised 393,722 cells from 145 sample entries, including 28,230 cells from 12 GSE214695 samples and 365,492 cells from 133 SCP259 sample entries. Because SCP259 contains repeated biopsies or tissue-fraction entries from the same individuals, sample and donor identities were retained separately, and repeated entries from the same participant were not treated as independent biological replicates in donor-level inference. The complete sample-to-donor mapping is provided in **Supplementary Table S2** and the public analysis repository.

### Cell type annotation and epithelial cell isolation

Cell types were annotated on the basis of cluster-level transcriptional profiles, canonical lineage markers, and available reference annotations. Major cell lineages included epithelial cells, fibroblasts, smooth muscle cells/pericytes, endothelial cells, myeloid cells, mast cells, T/NK cells, B cells, and plasma cells. Representative markers included EPCAM, KRT8, KRT18, and KRT19 for epithelial cells; COL1A1, COL1A2, DCN, and LUM for fibroblasts; ACTA2, TAGLN, RGS5, and PDGFRB for smooth muscle cells/pericytes; PLVAP, CLDN5, VWF, and RAMP2 for endothelial cells; LYZ, C1QA, C1QB, and FCER1G for myeloid cells; KIT, TPSAB1, TPSB2, and CPA3 for mast cells; NKG7, TRAC, CD3D, and KLRD1 for T/NK cells; MS4A1, CD79A, CD79B, and CD74 for B cells; and MZB1, JCHAIN, IGKC, and XBP1 for plasma cells. Annotation consistency was assessed using FeaturePlot and DotPlot visualization of representative marker expression.

Epithelial cells were independently reprocessed using 3,000 highly variable genes and 50 principal components. Harmony dimensions 1–30 were used for neighborhood construction and UMAP visualization, with clustering performed at a resolution of 0.6. Cell-cycle variation was not regressed during epithelial reclustering because proliferative state was itself a biological feature of interest. Epithelial states were annotated from cluster-level marker patterns and transcriptional characteristics. The ISC-like compartment was subsequently analyzed using dimensions 1–25, the ISC_snn graph, and a final clustering resolution of 0.3. The resulting ISC-like dataset comprised 9,306 cells from 33 donors and nine transcriptional subclusters. Stem/ISC-associated features were summarized using LGR5, ASCL2, OLFM4, SMOC2, SOX9, and PROM1.

### Reclassification of ISC-like epithelial cells and GART stratification

After isolating ISC-like epithelial cells, secondary clustering was performed to characterize transcriptional heterogeneity within this compartment. The ISC-like subset was reprocessed using the Seurat workflow for normalization, dimensionality reduction, neighborhood graph construction, clustering, and UMAP visualization. Predefined ISC and TA/proliferating signatures were scored using Seurat AddModuleScore and used to characterize the transcriptional features of the resulting subclusters.

For all revised analyses, GART status was defined directly from the corrected raw RNA count layer rather than from normalized expression values. Cells with a raw GART count of 0 were classified as GART-undetected, whereas cells with a raw GART count ≥1 were classified as GART-detected. A more stringent threshold of raw GART count ≥2 was prespecified as a sensitivity analysis. Cells with missing raw-count information were not coded as GART-undetected. The final ISC-like dataset contained 9,306 cells from 33 donors with recoverable GART raw counts.

### Donor-aware analysis of GART detection in ISC-like epithelial cells

To evaluate whether GART detection in ISC-like epithelial cells differed between UC and healthy controls while accounting for repeated sampling and technical covariates, donor-aware analyses were performed. For descriptive summaries, the GART-detected fraction was calculated for each donor as the number of ISC-like epithelial cells with raw GART count ≥1 divided by the total number of ISC-like epithelial cells contributed by that donor.

The primary inferential analysis used a binomial generalized linear mixed-effects model with single-cell GART detection status as the outcome. Disease condition, log1p-transformed UMI count, number of detected genes, S-phase score, G2/M-phase score, and cohort were included as fixed effects, and donor identity was included as a random intercept. This model therefore accounted for differences in transcript complexity and cell-cycle state while treating the donor, rather than the individual cell or sample entry, as the biological unit underlying inference.

Because some participants in SCP259 contributed multiple biopsy or tissue-fraction entries, an additional sensitivity model incorporated sample identity nested within donor identity. Cohort-specific models were also performed separately for GSE214695 and SCP259 to assess consistency between datasets. Sensitivity analyses further repeated the disease comparison using the more stringent raw-count definition of GART detection (raw GART count ≥2). Effect estimates are reported as odds ratios with 95% confidence intervals and two-sided P values.

### Differential expression analysis and gene set enrichment analysis

Functional transcriptional analyses comparing GART-detected and GART-undetected states were restricted to UC-derived ISC-like epithelial cells. To avoid treating individual cells as independent biological replicates, raw gene counts were aggregated within each donor according to GART detection status to generate donor-by-state pseudobulk profiles. Donors were eligible for the primary paired pseudobulk analysis when they contributed at least 20 ISC-like epithelial cells to both the GART-detected and GART-undetected states. Under this prespecified criterion, 11 paired donors from SCP259 were retained for differential expression analysis. GSE214695 did not contain sufficient paired cells to meet this criterion and was therefore not considered estimable for the paired pseudobulk analysis.

Differential expression was assessed using a paired donor design comparing GART-detected with GART-undetected pseudobulk profiles. Multiple-testing correction was performed using the Benjamini–Hochberg method. Genes were ranked according to the donor-paired differential-expression results and subjected to preranked gene set enrichment analysis. Hallmark, Gene Ontology Biological Process, and Reactome gene sets were evaluated (17–20). Positive normalized enrichment scores indicated preferential enrichment in the GART-detected state, whereas negative scores indicated preferential enrichment in the GART-undetected state. Enrichment P values were adjusted for multiple testing using the Benjamini–Hochberg method.

### Functional module scoring and cell cycle analysis

Functional module scores for purine biosynthesis, DNA replication, DNA repair, and mitotic cell-cycle processes were calculated from log-normalized RNA expression using Seurat AddModuleScore. Gene sets were derived from versioned Reactome, Gene Ontology, and MSigDB resources, and GART was excluded from the purine-biosynthesis module to avoid circular validation. Module scoring used an expressed-gene control pool with 24 expression bins and 100 control genes per feature set, using a fixed random seed of 20260902.

Associations between GART detection status and module scores were evaluated using donor-aware linear mixed-effects models. GART detection status, log1p-transformed UMI count, number of detected genes, S-phase score, G2/M-phase score, and cohort were included as fixed effects, with donor identity included as a random intercept. Sensitivity analyses additionally accounted for sample identity nested within donor and repeated the comparisons within G1, S, and G2/M cell-cycle strata.

Cell-cycle status was assigned using Seurat CellCycleScoring based on canonical S-phase and G2/M-phase gene sets. Cell-cycle distributions were summarized descriptively, while S-phase and G2/M-phase scores were incorporated as covariates in the donor-aware models described above.

### Comparison with other *de novo* purine-biosynthesis enzymes

To assess whether the observed GART-associated pattern reflected a GART-specific phenomenon or a broader alteration in *de novo* purine biosynthesis, parallel detection analyses were performed for PPAT, GART, PFAS, PAICS, ADSL, and ATIC in ISC-like epithelial cells. For each enzyme, detection status was defined from the corresponding raw RNA count layer using the same primary threshold applied to GART (raw count ≥1 versus 0). Disease-associated differences in detection were evaluated using donor-aware generalized linear mixed-effects models with adjustment for log1p-transformed UMI count, number of detected genes, S-phase score, G2/M-phase score, and cohort, with donor identity included as a random intercept. Effect estimates were summarized as odds ratios with 95% confidence intervals, and P values were adjusted for multiple testing using the Benjamini–Hochberg method.

### Pseudotime trajectory analysis

ISC-like epithelial cells were converted into a Monocle3-compatible cell data set for exploratory pseudotime analysis (21). The existing UMAP representation was used for trajectory visualization, and Monocle3 was used to learn the principal graph across the ISC-like epithelial manifold. The root state was selected using transcriptional information independent of GART expression or GART detection status, thereby avoiding incorporation of the expected GART direction into trajectory orientation.

Pseudotime values were extracted for cells assigned finite values. GART-detected and GART-undetected cells were visualized along the inferred state continuum using the raw-count-based GART definition described above. For donor-level inference, pseudotime was summarized separately for GART-detected and GART-undetected cells within donors contributing both states, and paired donor-level comparisons were performed. Cell-level density distributions and expression patterns along pseudotime were retained as descriptive visualizations rather than treated as independent biological replicates.

To assess the extent to which the inferred continuum depended on GART or proliferative features, the trajectory analysis was repeated after excluding GART and predefined cell-cycle-associated genes from the feature set. Associations between GART expression and pseudotime were compared between the primary and feature-exclusion trajectories. Pseudotime was interpreted as an exploratory transcriptional-state continuum and not as evidence of a temporal transition or lineage progression between GART-undetected and GART-detected cells.

### Exploratory scTenifoldKnk-based GART virtual perturbation analysis

An exploratory in silico network perturbation analysis was performed using scTenifoldKnk to examine transcriptional-network changes predicted after simulated GART knockout in UC-derived ISC-like epithelial cells (22). Raw RNA counts were used for network construction. Mitochondrial, ribosomal, hemoglobin, and other highly abundant technical or non-informative features were removed before network inference to reduce technical contributions to the reconstructed network.

Five independent computational runs were performed using different prespecified random seeds. In each run, GART was computationally perturbed and genes were ranked according to perturbation distance. GART itself was excluded from downstream interpretation of candidate perturbed genes. Genes ranked within the top 100 in all five runs were classified as robust recurrent candidates, whereas genes appearing within the top 100 in at least three of five runs were classified as recurrent candidates. Cross-run summaries included median rank, best rank, perturbation distance, and recurrence across independent runs.

Functional patterns were further summarized for predefined gene sets related to mitotic cell cycle, DNA replication, DNA repair, purine biosynthesis, and ISC-associated states. As a sensitivity analysis, the number of cells sampled for network construction was increased from 500 to 1,000 cells per run, and the resulting candidate-gene rankings were compared with those from the primary five-run analysis using 500 cells per run. Gene-level nominal P values returned by scTenifoldKnk were adjusted within each run using the Benjamini–Hochberg method. Because no permutation-based null procedure was performed, cross-run recurrence was used only as a measure of computational reproducibility.

### Donor-aware CellChat and LIANA+ cell–cell communication analysis

Cell–cell communication was explored using CellChat and LIANA+ with a focus on candidate incoming signals to GART-detected and GART-undetected ISC-like epithelial cells (23, 24). Analyses were performed separately at the donor level rather than by pooling cells across participants. Four predefined primary sender compartments were evaluated: myeloid cells, fibroblasts, endothelial cells, and T/NK cells. Other epithelial cells excluding the ISC-like receiver population were evaluated in sensitivity analyses. GART-detected and GART-undetected ISC-like cells were defined using the same raw-count criterion described above.

For the primary analysis, SCP259 donors were eligible when they contributed at least 20 cells to each receiver state and at least 20 cells to the corresponding sender compartment. A minimum expression proportion of 10% was used for ligand–receptor eligibility. CellChat analyses were performed on donor-specific objects using library-size normalization to 10,000 followed by log1p transformation and the human CellChat ligand–receptor database. LIANA+ analyses were performed independently on donor-specific expression objects generated from the same raw-count data and evaluated using its consensus ligand–receptor resource and multiple inference frameworks. LIANA+ used 100 internal permutations for its internal cell-level scoring procedure, whereas formal inference in this study was based on paired donor-level contrasts.

For each donor and sender–receiver combination, communication differences were defined as the inferred communication score toward GART-detected ISC-like cells minus that toward GART-undetected ISC-like cells. Statistical inference was performed on these within-donor contrasts using paired donor-level tests, and P values were adjusted across the tested interaction family using the Benjamini–Hochberg method. The proportion of donors showing concordant directional effects was also recorded. GSE214695 was subjected to the same eligibility criteria and workflow and was reported as not estimable when the prespecified minimum cell and donor requirements were not met.

Robustness analyses repeated the communication inference using the stricter GART detection threshold of raw count ≥2, minimum cell requirements of 10, 20, and 30 cells, and expression-proportion thresholds of 5%, 10%, and 20%. Leave-one-donor-out analyses were additionally used to evaluate directional stability. Communication analyses were interpreted as exploratory ligand–receptor associations and not as evidence of causal signaling or functional dependence.

### Analysis of SCENIC regulon activity

SCENIC was used to characterize transcriptional regulatory programs in the ISC-like epithelial compartment (25). Gene regulatory networks were inferred using GENIE3, followed by motif-enrichment–based regulon refinement, and regulon activity was quantified at the single-cell level using AUCell. Mitochondrial, ribosomal, hemoglobin, and other highly abundant non-informative genes were excluded before network inference to reduce technical contributions to the reconstructed regulatory networks.

Associations between GART detection status and regulon activity were evaluated using donor-aware linear mixed-effects models with AUCell score as the outcome. GART detection status, log1p-transformed UMI count, number of detected genes, S-phase score, G2/M-phase score, and cohort were included as fixed effects, with donor identity included as a random intercept. P values across regulons were adjusted using the Benjamini–Hochberg method.

### HGNN/SigXTalk prioritization of receptor–transcription factor–target paths

HGNN/SigXTalk was used as an exploratory approach to prioritize predicted receptor–transcription factor–target paths associated with the GART-detected ISC-like epithelial state (26). Predicted paths were filtered according to the HGNN prediction score, with a threshold of >0.75 used to retain higher-confidence candidate paths. Because the PRS-based downstream ranking step was not used in the final analysis, retained HGNN-predicted paths were further prioritized according to expression support within GART-detected ISC-like epithelial cells.

For each retained receptor–transcription factor–target path, expression support was calculated from the corresponding receptor, transcription factor, and target-gene expression in the receiver population. An expression-supported score was defined as the HGNN prediction label multiplied by the log1p-transformed mean expression of the receptor, transcription factor, and target gene. Candidate receptor, transcription-factor, and target nodes were then ranked according to these expression-supported paths.

### Spatial transcriptomics analysis

Spatial transcriptomic analysis was performed using the publicly available GSE189184 dataset (27). Seven retained colonic tissue sections, including two healthy controls and five UC samples, corresponded to seven independent donors. Processed spatial transcriptomic data were analyzed in Seurat, and the Spatial assay was normalized using LogNormalize when normalized expression values were not already available.

An external GART-associated epithelial program was derived independently from the SCP259 single-cell dataset using donor-blocked training and a nearest-centroid approach and then projected onto the spatial transcriptomic data. Within each tissue section, epithelial regions were defined using a composite EPCAM/KRT8/KRT18/KRT19 expression score, and spots within the top 40% of this epithelial score were retained as the primary epithelial ROI. Spatial analyses were performed at raw-spot resolution.

Within the epithelial ROI, spots with a corrected raw GART count of 0 were classified as GART-undetected and those with a raw GART count ≥1 as GART-detected. For each donor, the mean external-program score was calculated separately for GART-detected and GART-undetected spots, and the within-donor difference was used as the primary spatial endpoint. Donors were treated as the independent biological units. Statistical inference was performed using a two-sided exact paired sign-flip test, with donor-level bootstrap resampling used to estimate the 95% confidence interval. The complete section-to-donor mapping, external-program definition, epithelial ROI specification, donor-level spatial endpoints, and analysis parameters are provided in **Supplementary Table S6**.

### Multiplex immunofluorescence staining in human tissues

To examine the tissue-level localization of the GART-associated epithelial state, multiplex immunofluorescence was performed on human colonic mucosal sections using three staining panels. Participant numbers differed among panels: the GART/MIF/CD74 panel included 18 healthy controls and 25 patients with UC; the GART/MYBL2/Ki67 panel included 20 healthy controls and 20 patients with UC; and the GART/EPCAM/OLFM4 panel included 53 healthy controls and 42 patients with UC. The participant was treated as the independent biological and statistical unit for each panel. Available participant characteristics, including age, sex, biopsy site, UC extent, disease activity, and treatment exposure, are summarized in **Supplementary Table S7**.

Formalin-fixed paraffin-embedded sections were deparaffinized in xylene, rehydrated through graded ethanol, and subjected to heat-mediated antigen retrieval in citrate buffer (pH 6.0) for 30 min. Endogenous peroxidase activity was blocked with 3% hydrogen peroxide for 10 min, followed by blocking with 3% BSA for 30 min.

Multiplex staining was performed using a sequential tyramide signal amplification (TSA) procedure. In each staining cycle, sections were incubated with the corresponding rabbit primary antibody overnight at 4 °C, followed by a ready-to-use polymer-HRP goat anti-rabbit reagent for 30 min at room temperature and the corresponding tyramide fluorophore for 10 min. After completion of the first and second TSA cycles, sections were heated in citrate antigen-retrieval buffer (pH 6.0) for 30 min to remove the bound primary and HRP-conjugated secondary antibodies before the subsequent staining cycle.

The staining sequence was GART–CY3, OLFM4–CY5, and EPCAM–Fluorescein for the GART/EPCAM/OLFM4 panel; GART–CY3, MYBL2–CY5, and Ki67–Fluorescein for the GART/MYBL2/Ki67 panel; and GART–CY3, MIF–CY5, and CD74–Fluorescein for the GART/MIF/CD74 panel. Nuclei were counterstained with DAPI for 10 min.

Slides were scanned using an Olympus VS200 digital slide scanner with a 20× objective. DAPI, Fluorescein, CY3, and CY5 signals were acquired using dedicated narrow-band optical filter sets. Detailed antibody information, TSA reagents, staining sequence, stripping conditions, optical filter specifications, and acquisition information are provided in **Supplementary Table S8**.

Detailed historical information concerning field/crypt selection, image-acquisition blinding, quantitative-analysis blinding, and threshold-setting procedures for the archived human multiplex-immunofluorescence analyses could not be fully reconstructed. These unavailable procedural details were therefore not inferred.

### DSS-induced colitis mouse model

A 2.5% DSS-induced acute colitis model was established for tissue-level analysis (28). Male wild-type C57BL/6J mice, aged 6–8 weeks and weighing 18–22 g, were purchased from Jiangsu Huachuang Xinnuo Pharmaceutical Technology Co., Ltd. (Animal Certificate No. B202503050715). Mice were housed under specific pathogen-free conditions at the Affiliated Hospital of Nanjing University of Chinese Medicine at 20–25 °C and 40%–60% relative humidity under a 12-h light/dark cycle and were acclimatized for 7 days before the experiment.

After acclimatization, mice were randomly allocated to a healthy control group or a DSS group (n = 6 per group). Control mice received purified water, whereas DSS-treated mice received drinking water containing 2.5% DSS for 7 days, followed by purified water for 3 days. Body weight, stool consistency, and fecal blood were recorded daily, and the disease activity index (DAI) was calculated throughout the experiment.

On day 11, mice were anesthetized with sodium pentobarbital for terminal blood collection and subsequently euthanized by intraperitoneal overdose of sodium pentobarbital (150 mg/kg). Death was confirmed by the absence of respiration and heartbeat before tissue collection. Colon tissues were harvested for measurement of colon length and subsequent histopathological, immunohistochemical, and multiplex immunofluorescence analyses.

All six mice per group contributed to the longitudinal and systemic analyses. For imaging analyses, three of the six animals in each group were randomly selected before staining and image quantification. Selection was performed without reference to disease phenotype, DAI, histological severity, staining intensity, or post-acquisition image quality. The remaining animals were not selected for these imaging endpoints and were not treated as experimental exclusions.

### Complete blood count and serum inflammatory marker testing

At terminal collection on day 11, retro-orbital blood was obtained under deep anesthesia and divided for hematological and serum analyses. Anticoagulated whole blood was used for complete blood count analysis, including hemoglobin (HGB), hematocrit (HCT), platelet count (PLT), granulocyte percentage (Gran%), and white blood cell count (WBC). All six mice per group were included in these analyses.

For serum preparation, blood was allowed to clot at room temperature for 1 h and centrifuged at 3,000 rpm for 15 min. Serum concentrations of interleukin-1β (IL-1β), IL-6, IL-17A, tumor necrosis factor-α (TNF-α), and IL-10 were measured using commercial ELISA kits according to the manufacturers’ instructions. All six mice per group were included in the serum analyses. Information on the ELISA kits is provided in **Supplementary Table S9**.

### Histopathology and immunohistochemistry

Colon tissues were fixed in 4% paraformaldehyde, paraffin-embedded, sectioned, and stained with hematoxylin and eosin (H&E). Histopathological injury was evaluated according to inflammatory-cell infiltration, epithelial damage, crypt loss or destruction, and disruption of mucosal architecture.

Immunohistochemistry was performed to assess the epithelial barrier-associated proteins ZO-1, Claudin-1, and Occludin. Paraffin sections were deparaffinized, rehydrated through graded ethanol, subjected to antigen retrieval and blocking, and incubated with the corresponding primary antibodies overnight at 4 °C. Sections were subsequently incubated with an HRP-conjugated secondary antibody, developed with 3,3′-diaminobenzidine (DAB), and counterstained with hematoxylin.

Bright-field images were acquired using consistent imaging settings for each marker. Immunohistochemical quantification was performed at the animal level. Each plotted point therefore represents one individual mouse, and the mouse was treated as the independent biological and statistical unit. Antibody manufacturers, catalog numbers, and working dilutions are provided in **Supplementary Table S9**.

### Multicolor immunofluorescence in mouse tissues

Multiplex immunofluorescence was performed on colonic tissues from the DSS-induced acute colitis model to examine the tissue-level distribution of GART in relation to proliferative, ISC-like, inflammatory-context, and purine-metabolic markers. Four staining panels were evaluated: GART/MYBL2/Ki67, GART/EPCAM/OLFM4, GART/MIF/CD74, and GART/PAICS/PCNA. Three mice per group were analyzed for these imaging endpoints.

Formalin-fixed paraffin-embedded sections were processed using the same sequential tyramide signal amplification (TSA) workflow described above for human tissues. Briefly, each staining cycle consisted of incubation with one rabbit primary antibody overnight at 4 °C, followed by polymer-HRP goat anti-rabbit reagent for 30 min and deposition of the corresponding tyramide fluorophore for 10 min. After the first and second cycles, sections were subjected to citrate-based heat treatment (pH 6.0, 30 min) to remove the bound primary antibody and HRP-conjugated secondary reagent before the next staining cycle. The staining sequences were GART–CY3 → MYBL2–CY5 → Ki67–Fluorescein, GART–CY3 → OLFM4–CY5 → EPCAM–Fluorescein, GART–CY3 → MIF–CY5 → CD74–Fluorescein, and GART–CY3 → PAICS–CY5 → PCNA–Fluorescein. Nuclei were counterstained with DAPI.

Slides were scanned using an Olympus VS200 digital slide scanner with a 20× objective, and consistent acquisition settings were applied within each staining panel. Quantitative endpoints included GART fluorescence intensity within crypt or epithelial regions, MYBL2 nuclear fluorescence intensity, the proportion of Ki67-positive cells, OLFM4 fluorescence intensity within EPCAM-positive crypt epithelium, CD74-positive cell density and MIF-high mucosal area in relation to GART-positive regions, PAICS fluorescence intensity, the proportion of PCNA-positive cells, and the spatial overlap between GART and PAICS. Detailed antibody information, TSA reagents, staining sequences, stripping conditions, and optical acquisition parameters are provided in **Supplementary Table S8**.

### Quantification of immunofluorescence images

Quantitative image analysis was performed separately for each multiplex staining panel using the corresponding marker-specific endpoints. Fluorescence-based measurements were summarized as fluorescence intensity, positive-cell proportion, cell density, or spatial proximity/overlap metrics, as appropriate for each panel. The principal endpoints included GART fluorescence intensity in crypt or epithelial regions, OLFM4 fluorescence intensity within EPCAM-positive epithelium, MYBL2 nuclear fluorescence intensity, the proportions of Ki67-positive and PCNA-positive cells, CD74-positive cell density, MIF-high mucosal area, PAICS fluorescence intensity, and spatial proximity or overlap between GART-stained regions and the corresponding markers.

For human tissues, each participant was treated as an independent biological replicate and statistical unit; for mouse tissues, each mouse was treated as an independent biological replicate and statistical unit.

### Statistical analysis

Statistical analyses were performed using R and GraphPad Prism. All hypothesis tests were two-sided, and a P value <0.05 was considered statistically significant unless otherwise specified. Summary data are presented as mean ± SD unless otherwise indicated, and effect estimates are accompanied by 95% confidence intervals where applicable.

Analysis-specific statistical models, covariates, donor structures, and multiple-testing procedures for the transcriptomic and spatial analyses are described in the corresponding Methods subsections above.

For human multiplex immunofluorescence analyses, each participant was treated as an independent biological and statistical unit. Healthy-control and UC groups were compared using two-sided Mann–Whitney U tests for endpoints analyzed nonparametrically. CD74-positive cell density, Ki67-positive cell percentage, and GART/Ki67 double-positive cell percentage were analyzed using two-sided Welch’s unpaired t tests. Results are reported as median (interquartile range) with Hodges–Lehmann UC-minus-HC location shifts and 95% confidence intervals for Mann–Whitney analyses or mean ± SD with UC-minus-HC mean differences and Welch–Satterthwaite 95% confidence intervals for Welch analyses.

For mouse two-group endpoints, including complete blood count parameters, serum cytokines, barrier-protein immunohistochemistry, and multiplex immunofluorescence measurements, comparisons were performed using two-sided unpaired Student’s t-tests with equal variances. P values were adjusted within each prespecified endpoint family using the Benjamini–Hochberg method.

Longitudinal body-weight and DAI measurements were analyzed using mixed-effects models including time, group, and the group × time interaction.

## Results

### Bulk transcriptomic evidence and construction of a human UC single-cell atlas

We first evaluated GART expression across four independent human intestinal mucosal transcriptomic cohorts: GSE24287, GSE36807, GSE37283, and GSE87466. All four cohorts showed positive effect estimates for GART expression in UC relative to healthy controls, with Hedges’ g values of 0.656, 1.287, 1.484, and 0.596, respectively. Random-effects meta-analysis yielded a pooled Hedges’ g of 0.743 (95% CI, 0.239–1.248; P = 0.0183), with no detected between-cohort heterogeneity (I² = 0%; **Figure 1**; **Supplementary Figure S1**). These bulk-tissue data therefore supported an overall association between UC and higher GART expression, without distinguishing the cellular source or the contribution of inflammation and epithelial turnover.

**Figure 1:**
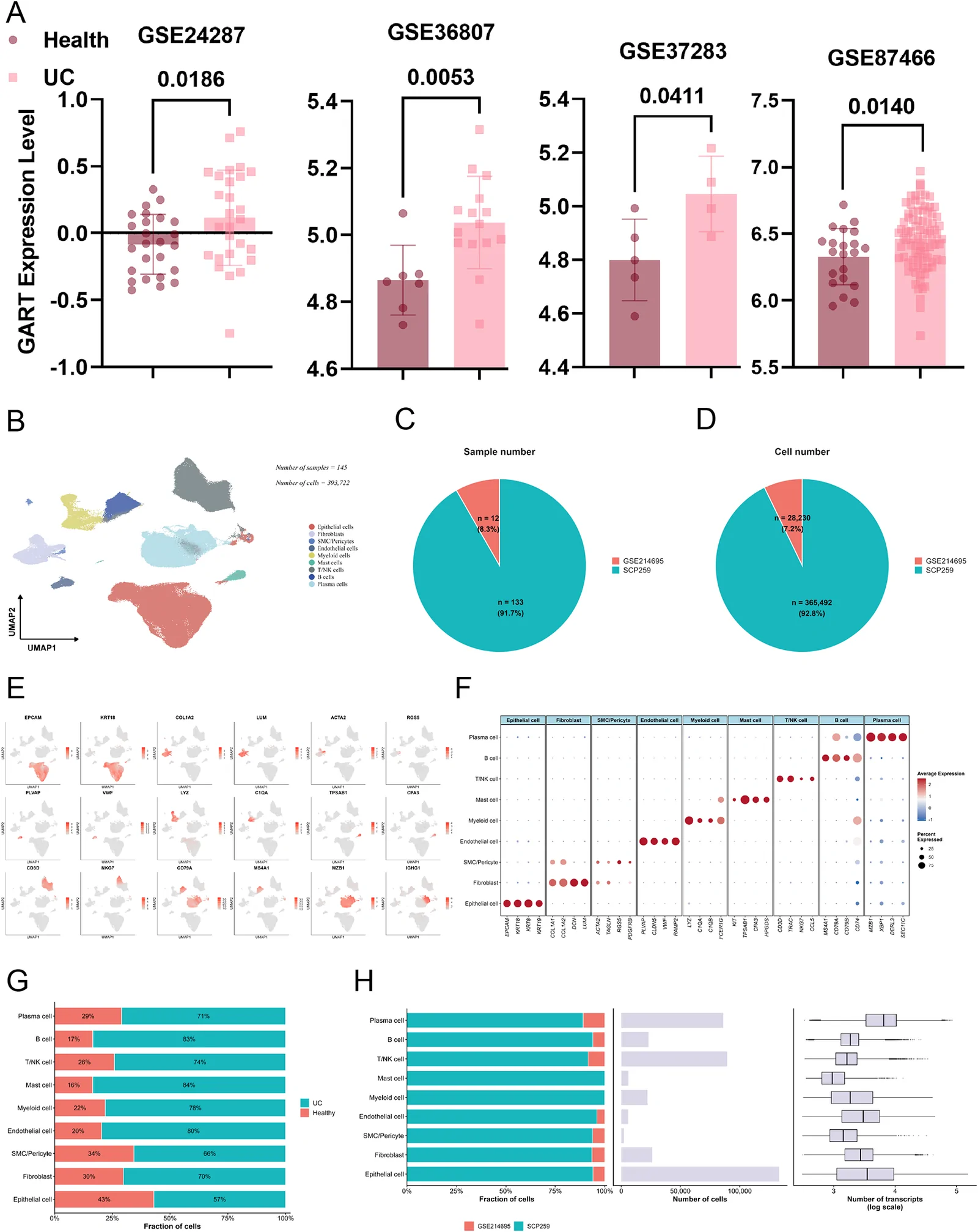
Bulk transcriptomic evidence and construction of a human UC single-cell atlas. **(A)** GART expression in healthy control and UC mucosal samples across GSE24287, GSE36807, GSE37283, and GSE87466. **(B)** UMAP visualization of the integrated human colonic mucosal scRNA-seq atlas showing major cell lineages. **(C, D)** Dataset composition according to sample entries and cell numbers for GSE214695 and SCP259. **(E)** FeaturePlot visualization of representative lineage markers. **(F)** DotPlot showing canonical markers used for cell-type annotation. **(G)** Disease-state composition across major lineages. **(H)** Dataset-source contribution, cell numbers, and transcript complexity across major cell types. Bulk cohort sample sizes were HC/UC = 25/27 (GSE24287), 7/15 (GSE36807), 5/4 (GSE37283), and 21/87 (GSE87466). The integrated single-cell atlas contained 393,722 cells from 145 sample entries; atlas-level cell displays are descriptive.

We next integrated two publicly available human colonic scRNA-seq datasets, GSE214695 and SCP259. After quality control and preprocessing, the integrated atlas contained 393,722 cells from 145 sample entries, including 28,230 cells from 12 GSE214695 samples and 365,492 cells from 133 SCP259 sample entries (**Figure 1B**). Because SCP259 contains repeated biopsy or tissue-fraction entries from the same individuals, these entries were retained separately for atlas construction but were not treated as independent biological replicates in downstream donor-level inference.

Major cell populations were annotated according to canonical lineage markers, including EPCAM, KRT8, KRT18, and KRT19 for epithelial cells; COL1A1, COL1A2, DCN, and LUM for fibroblasts; ACTA2, TAGLN, RGS5, and PDGFRB for smooth muscle cells/pericytes; PLVAP, CLDN5, VWF, and RAMP2 for endothelial cells; LYZ, C1QA, C1QB, and FCER1G for myeloid cells; KIT, TPSAB1, TPSB2, and CPA3 for mast cells; NKG7, TRAC, CD3D, and KLRD1 for T/NK cells; MS4A1, CD79A, CD79B, and CD74 for B cells; and MZB1, JCHAIN, IGKC, and XBP1 for plasma cells (**Figure 1E**).

Examination of disease-status and dataset composition showed that cells from both healthy and UC samples were represented across the major lineages, although their relative contributions varied by condition and dataset (**Figure 1G**). This integrated atlas provided the basis for subsequent epithelial-subset analyses.

### GART detection defines a heterogeneous state within the ISC-like epithelial compartment, with cohort-dependent UC associations

We next isolated epithelial cells and annotated major epithelial states, including absorptive epithelial cells, goblet cells, regenerative epithelial cells, ISC-like epithelial cells, TA/proliferating cells, BEST4-positive epithelial cells, rare epithelial cells, and low-confidence/other epithelial cells (**Figure 2**). Canonical epithelial markers supported the annotation of these populations (**Figure 2**).

**Figure 2:**
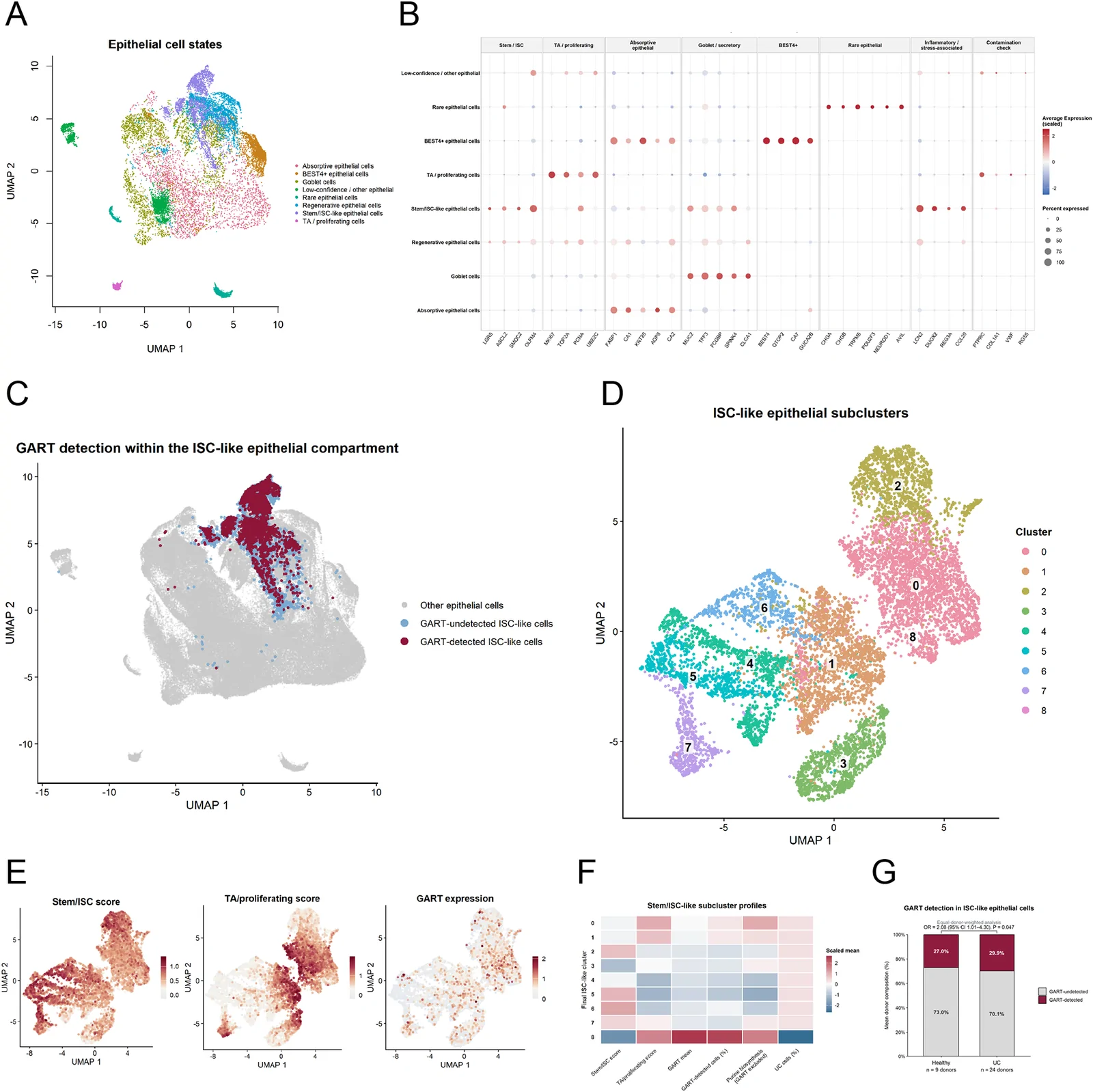
GART detection defines a heterogeneous state within the ISC-like epithelial compartment. **(A)** UMAP visualization of epithelial cell states after epithelial-cell isolation and reclustering. **(B)** DotPlot of representative markers used for epithelial-state annotation. **(C)** Distribution of GART-undetected and GART-detected ISC-like epithelial cells within the epithelial atlas. GART status was defined using corrected raw counts (undetected, count = 0; detected, count ≥1). **(D)** UMAP visualization of nine ISC-like epithelial subclusters. **(A)** Stem/ISC score, TA/proliferating score, and GART expression across ISC-like cells. **(F)** Subcluster-level transcriptional profiles. **(G)** Equal-donor-weighted comparison of GART detection in healthy and UC donors. The ISC-like subset contained 9,306 cells from 33 donors (9 healthy and 24 UC); donor was the biological unit for donor-level comparisons.

We then focused on the ISC-like epithelial compartment and examined GART detection within this population (**Figure 2**). Secondary analysis identified nine transcriptionally distinct ISC-like subclusters (**Figure 2**). Stem/ISC and TA/proliferating signature scores, together with GART expression and detection metrics, varied across these subclusters, demonstrating substantial transcriptional heterogeneity within the ISC-like compartment (**Figure 2E**).

The final ISC-like dataset comprised 9,306 cells from 33 donors with recoverable raw GART counts. GART status was defined as undetected (raw count = 0) or detected (raw count ≥1), replacing the previous negative/low/high classification. An equal-donor-weighted comparison showed a modestly higher GART-detected fraction in UC than in healthy controls (29.9% vs 27.0%; OR = 2.08, 95% CI 1.01–4.30; P = 0.047; **Figure 2**). However, in the prespecified covariate-adjusted donor-aware mixed-effects model, the overall disease association was attenuated and did not reach statistical significance (OR = 2.41, 95% CI 0.85–6.86; P = 0.0987; **Supplementary Table S2**). Cohort-specific analyses further showed that the association was not uniform: no significant difference was observed in GSE214695 (OR = 0.69, 95% CI 0.11–4.27; P = 0.6942), whereas SCP259 showed a higher probability of GART detection in UC (OR = 4.90, 95% CI 1.08–22.20; P = 0.0393; **Supplementary Figure S2A**; **Supplementary Table S2**). Donor-level GART-detected fractions for the two cohorts are shown separately in **Supplementary Figure S2B**. Thus, the UC-associated difference in GART detection was cohort dependent rather than consistently observed across datasets.

### GART-detected ISC-like epithelial cells exhibit proliferative and purine-metabolic programs

Within UC-derived ISC-like epithelial cells, GART-detected and GART-undetected cells showed heterogeneous distributions across the transcriptional landscape (**Figure 3**). To evaluate transcriptional differences without treating individual cells as independent biological replicates, differential-expression analysis was performed using paired donor-level pseudobulk profiles. Eleven SCP259 donors contributed sufficient numbers of both GART-detected and GART-undetected ISC-like cells for the prespecified paired analysis, whereas GSE214695 did not meet the corresponding paired-cell requirement. Donor-paired differential-expression analysis identified higher expression of multiple proliferation- and DNA-replication-associated genes in the GART-detected state, including GINS2, FEN1, TK1, MCM7, PCNA, TYMS, MCM6, and PAICS (**Figure 3**; **Supplementary Table S3**).

**Figure 3:**
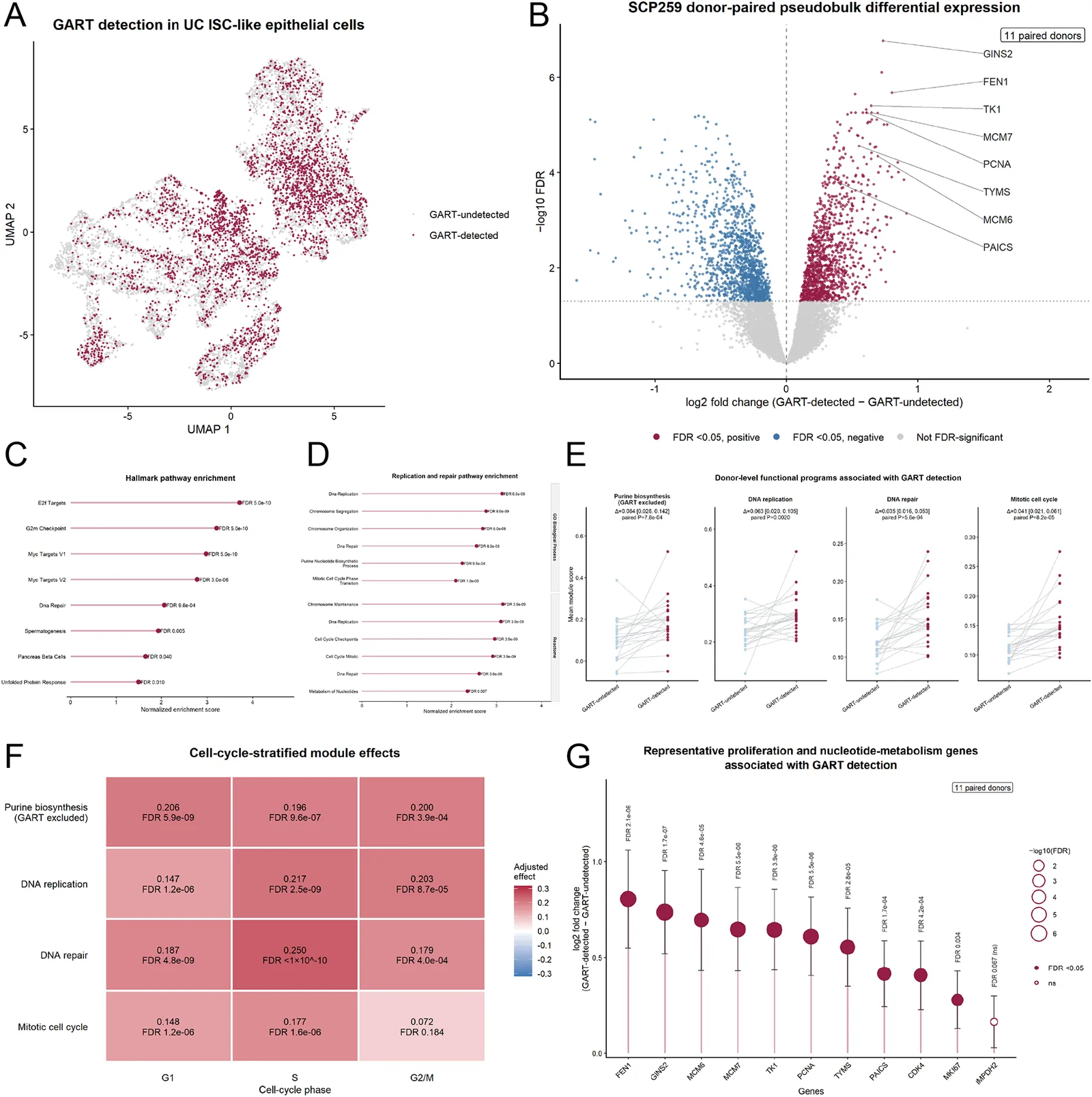
GART-detected ISC-like epithelial cells exhibit proliferative and purine-metabolic programs. **(A)** UMAP visualization of GART-undetected and GART-detected UC-derived ISC-like epithelial cells. **(B)** SCP259 donor-paired pseudobulk differential-expression analysis in 11 paired UC donors; representative proliferation- and replication-associated genes are labeled. **(C)** Hallmark pathway enrichment based on the same 11 paired UC donors. **(D)** Representative replication- and DNA-repair pathway enrichment based on the same 11 paired UC donors. **(E)** Donor-level comparisons of GART-excluded purine-biosynthesis, DNA-replication, DNA-repair, and mitotic-cell-cycle module scores across 23 donors contributing both GART states (21 UC and 2 healthy-control donors). **(F)** Cell-cycle-stratified covariate-adjusted module effects across the broader ISC-like atlas (G1, n = 30 donors; S, n = 25 donors; G2/M, n = 21 donors). **(G)** Donor-paired effects for representative proliferation- and nucleotide-metabolism-associated genes in the same 11 paired UC donors. FDR-adjusted values are shown where applicable. Donor was the biological and inferential unit for donor-aware comparisons.

Gene set enrichment analysis based on the donor-paired pseudobulk results showed positive enrichment of proliferation- and genome-maintenance-related programs, including E2F targets, G2/M checkpoint, DNA replication, and DNA repair pathways in the GART-detected state (**Figure 3C**; **Supplementary Table S3**). These findings indicated that the transcriptional differences associated with GART detection extended beyond individual marker genes and involved broader proliferative and nucleotide-demand programs.

Across the broader ISC-like epithelial atlas, we next examined purine biosynthesis, DNA replication, DNA repair, and mitotic cell-cycle module scores. GART was excluded from the purine-biosynthesis module to avoid circularity. Donor-level summaries from 23 donors contributing both GART states showed higher scores for all four programs in GART-detected than in GART-undetected cells (**Figure 3**; **Supplementary Table S3**), and covariate-adjusted mixed-effects analyses supported positive associations between GART detection and these programs. When the analyses were repeated separately within cell-cycle phases, the adjusted effects remained positive across all strata (G1, n = 30 donors; S, n = 25; G2/M, n = 21; **Figure 3**; **Supplementary Figure S3**; **Supplementary Table S3**). Most associations remained significant after multiple-testing correction, although the mitotic-cell-cycle association within the G2/M phase did not remain FDR-significant (adjusted effect = 0.072; FDR = 0.184).

Representative proliferation- and nucleotide-metabolism-associated genes also showed positive donor-paired effects in the GART-detected state (**Figure 3**). However, parallel analyses of other *de novo* purine-biosynthesis enzymes, including PPAT, PFAS, PAICS, ADSL, and ATIC, did not support a uniquely GART-specific disease-associated alteration (**Supplementary Table S3**). Together, these findings characterize GART detection as a feature of a broader proliferative and purine-metabolic ISC-like epithelial state rather than evidence of a GART-specific regenerative program.

### Pseudotime sensitivity and exploratory virtual perturbation characterize the GART-associated epithelial state

Exploratory pseudotime analysis was performed to examine the position of GART-detected cells along the ISC-like epithelial transcriptional continuum. Using a GART-independent root, GART-detected and GART-undetected cells showed different distributions along the inferred continuum (**Figure 4A**). At the donor level, GART-detected cells showed a modest shift in median continuum position relative to GART-undetected cells across 23 paired donors (paired Wilcoxon P = 0.0235; **Figure 4**). After excluding GART and predefined cell-cycle genes from trajectory construction, the reconstructed feature-exclusion trajectory is shown in **Supplementary Figure S4**, and the correlation between GART expression and continuum position was markedly reduced from Spearman ρ = 0.199 to ρ = 0.037 (**Figure 4**). These sensitivity results indicate that a substantial component of the apparent pseudotime association was related to proliferative and cell-cycle structure.

**Figure 4:**
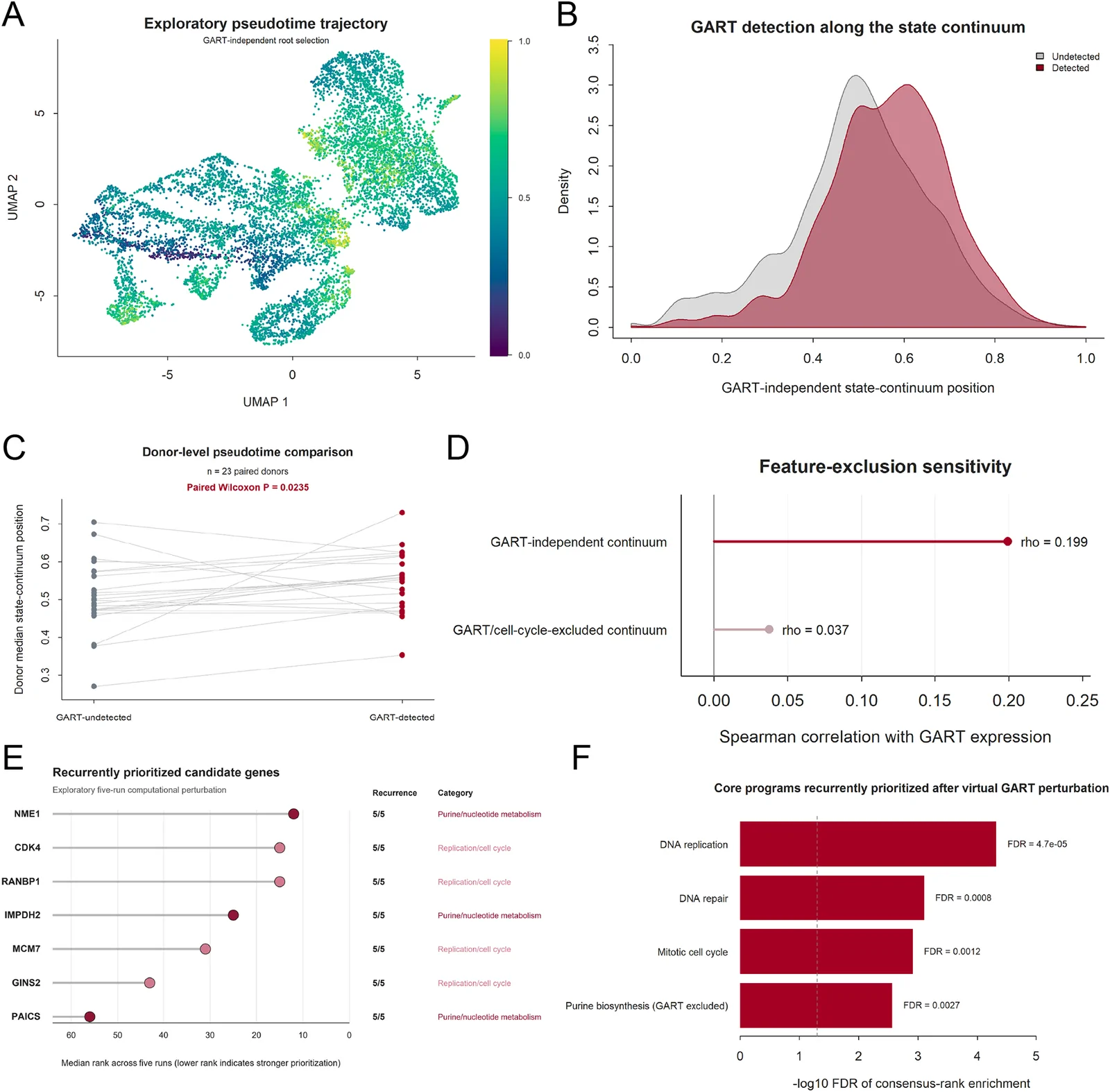
Pseudotime sensitivity and exploratory virtual perturbation characterize the GART-associated state.**(A)** Exploratory ISC-like epithelial state continuum constructed using a GART-independent root. **(B)** Density distributions of GART-undetected and GART-detected cells along the inferred continuum. **(C)** Donor-level comparison of median continuum positions across 23 paired donors. **(D)** Sensitivity analysis showing the correlation between GART expression and continuum position before and after exclusion of GART and predefined cell-cycle genes. **(E)** Seven representative robust recurrent candidate genes prioritized within the top 100 across all five scTenifoldKnk runs. **(F)** Core DNA-replication, DNA-repair, mitotic-cell-cycle, and GART-excluded purine-biosynthesis programs recurrently prioritized after virtual GART perturbation. The trajectory included 9,306 ISC-like cells from 33 donors; the donor-level comparison in **(C)** included 23 paired donors. Cell-level trajectory displays are descriptive, and donor was the biological unit for formal paired inference.

We next used scTenifoldKnk as an exploratory in silico network-perturbation approach to characterize candidate transcriptional features associated with simulated GART perturbation in UC ISC-like epithelial cells. Five independent computational runs were performed using prespecified random seeds. Genes ranked within the top 100 in all five runs were classified as robust recurrent candidates. Fifteen genes met the robust recurrence criterion; seven representative robust candidates—NME1, CDK4, RANBP1, IMPDH2, MCM7, GINS2, and PAICS—were associated primarily with nucleotide metabolism, DNA replication, and cell-cycle regulation (**Figure 4**; **Supplementary Table S4**).

At the pathway level, DNA replication, DNA repair, mitotic cell cycle, and GART-excluded purine biosynthesis were recurrently prioritized after virtual GART perturbation (**Figure 4**). Sensitivity analyses using alternative network-construction sampling settings are provided in **Supplementary Table S4**. Because scTenifoldKnk represents perturbation of an inferred network rather than direct experimental manipulation, and no explicit permutation-based null procedure was used, these findings were interpreted as hypothesis-generating network associations rather than evidence that GART is functionally required for epithelial proliferation or repair.

### GART-detected ISC-like cells exhibit distinct microenvironmental and transcriptional features

To explore candidate microenvironmental features associated with the GART-detected ISC-like state, we performed donor-aware cell–cell communication analyses focusing on the MIF/CD74 interaction family. Across 11 paired donors, CellChat showed positive MIF/CD74 communication-score differences toward GART-detected relative to GART-undetected ISC-like cells across the evaluated myeloid, fibroblast, endothelial, and T/NK sender compartments (BH FDR = 0.008275; **Figure 5**). LIANA+ consensus analysis showed a concordant directional pattern for MIF/CD74-family communication in the same donor set (BH FDR = 0.01020; **Figure 5**). The donor-aware communication landscape further illustrated positive MIF/CD74-family differences across these sender compartments (**Figure 5**). Because CellChat and LIANA+ were applied to the same underlying expression atlas, their concordance was interpreted as computational consistency rather than independent validation. These analyses therefore support a candidate MIF/CD74-associated communication context but do not establish causal regulation of GART.

**Figure 5:**
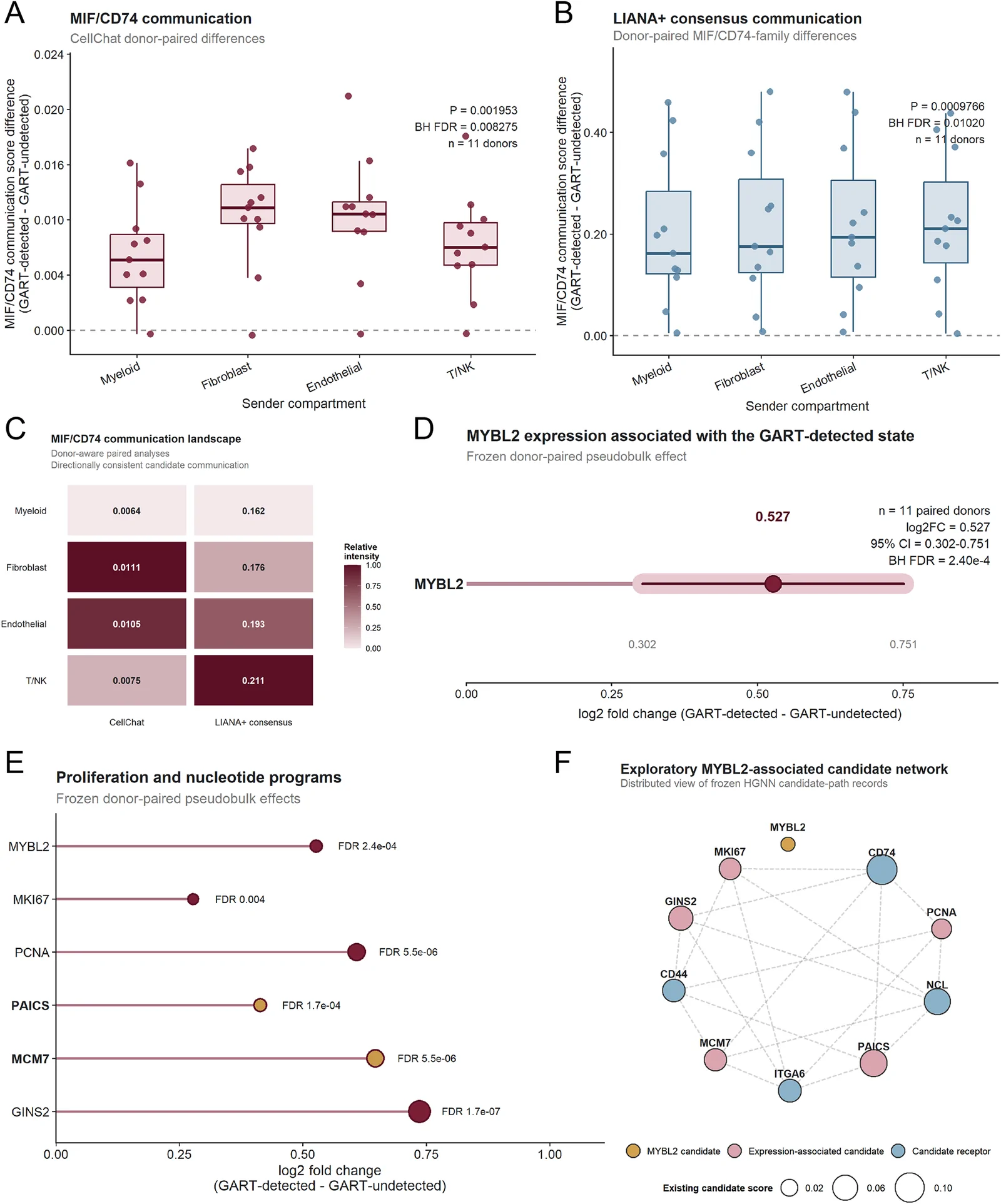
GART-detected ISC-like cells exhibit distinct microenvironmental and transcriptional features. **(A)** Donor-paired CellChat MIF/CD74 communication-score differences toward GART-detected versus GART-undetected ISC-like cells across sender compartments. **(B)** LIANA+ consensus MIF/CD74-family communication differences across the same sender compartments. **( C)** Donor-aware MIF/CD74 communication landscape showing directionally consistent candidate communication across CellChat and LIANA+. **(D)** Donor-paired pseudobulk MYBL2 expression difference between GART-detected and GART-undetected states. **(E)** Donor-paired effects for representative proliferation- and nucleotide-metabolism-associated genes. **(F)** Exploratory HGNN/SigXTalk-derived MYBL2-associated candidate network containing receptor and expression-associated candidate nodes. Primary donor-level communication and pseudobulk comparisons in **(A–E)** used 11 paired SCP259 donors; donor was the inferential unit. Panel **(F)** is an exploratory predicted network.

Donor-paired pseudobulk analysis showed higher MYBL2 expression in the GART-detected state (log2FC = 0.527, 95% CI 0.302–0.751; BH FDR = 2.40 × 10^-4^; n = 11 paired donors; **Figure 5**). Positive donor-paired effects were also observed for representative proliferation- and nucleotide-metabolism-associated genes, including MKI67, PCNA, PAICS, MCM7, and GINS2 (**Figure 5**). However, donor-aware SCENIC analysis after adjustment for technical and cell-cycle covariates did not support interpreting MYBL2 as an independently activated GART-specific regulon. MYBL2 was therefore considered a proliferation-associated transcriptional feature of the GART-detected state rather than a GART-specific regulator (**Supplementary Table S5**).

HGNN/SigXTalk was subsequently used as an exploratory approach to prioritize predicted receptor–transcription factor–target paths supported by expression in GART-detected ISC-like epithelial cells. The resulting candidate network highlighted receptor nodes including CD74, NCL, ITGA6, and CD44, together with MYBL2 and proliferation/nucleotide-metabolism-associated candidates including MKI67, GINS2, MCM7, PAICS, and PCNA (**Figure 5**; **Supplementary Table S5**). These predicted paths were interpreted as hypothesis-generating associations rather than evidence of a causal signaling hierarchy or a direct MYBL2–GART regulatory relationship.

### Spatial transcriptomics supports regional co-enrichment of the GART-associated epithelial program

We next examined the spatial organization of the GART-associated epithelial state using seven retained sections from GSE189184, comprising two healthy controls and five UC samples, with each section corresponding to an independent donor. Representative spatial maps showed heterogeneous localization of GART transcripts within colonic mucosa (**Figure 6**), with GART distributions across all seven retained sections shown in **Supplementary Figure S5A**. An independently derived single-cell GART-associated epithelial program was then projected onto the spatial data, and epithelial regions were defined within each section using an outcome-independent EPCAM/KRT8/KRT18/KRT19-based epithelial score (**Figure 6**). The corresponding external-program distributions across all seven sections are shown in **Supplementary Figure S5B**.

**Figure 6:**
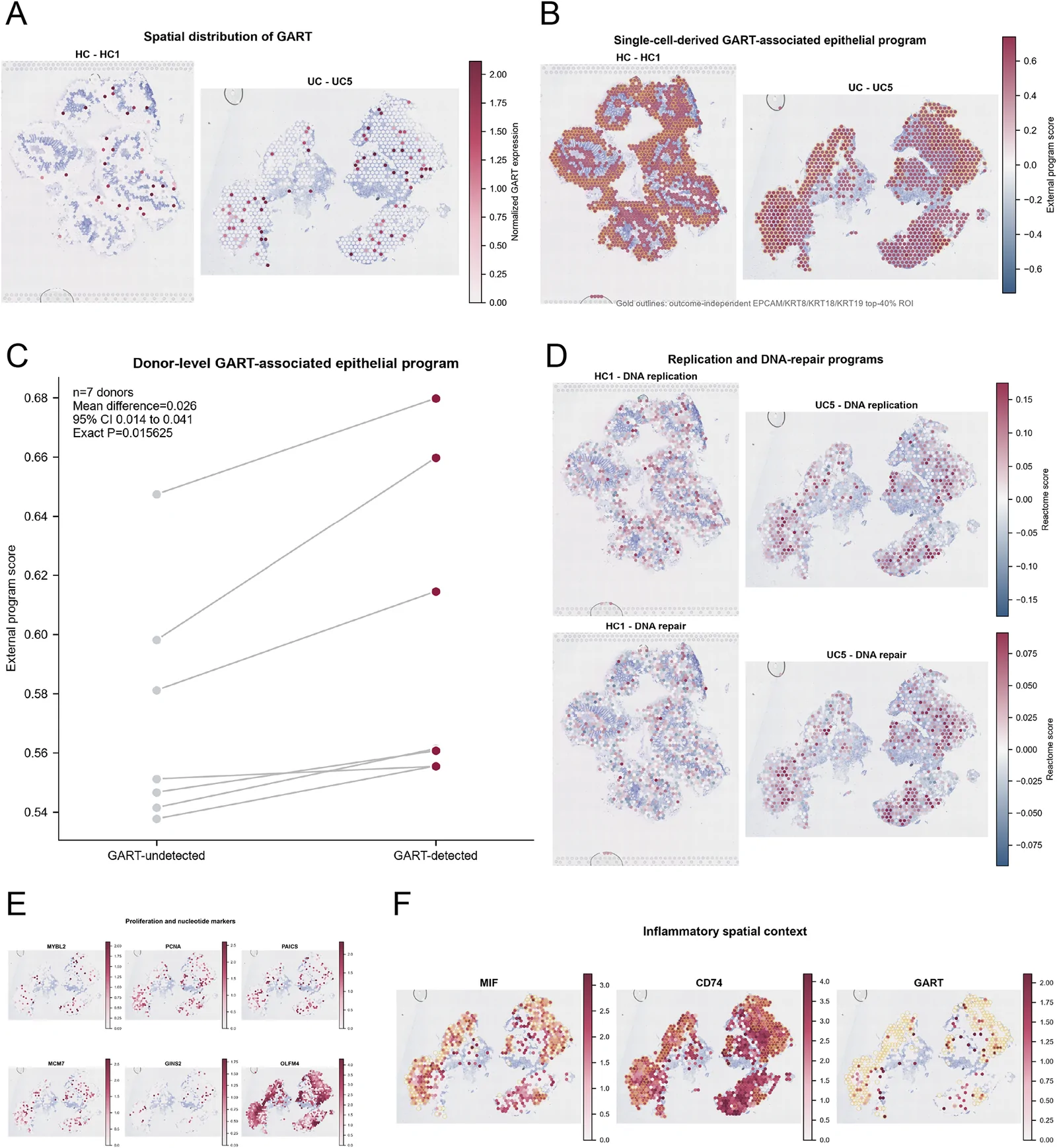
Spatial co-enrichment of the GART-associated epithelial program. **(A)** Representative spatial distribution of GART transcripts in healthy and UC colonic sections. **(B)** Projection of the independently derived single-cell GART-associated epithelial program onto spatial data; gold outlines indicate the outcome-independent EPCAM/KRT8/KRT18/KRT19 top-40% epithelial ROI. **(C)** Donor-level external-program scores in GART-undetected and GART-detected epithelial regions across seven independent donors. **(D)** Spatial distributions of DNA-replication and DNA-repair programs. **(E)** Spatial expression of MYBL2, PCNA, PAICS, MCM7, GINS2, and OLFM4. **(F)** Spatial distributions of MIF, CD74, and GART illustrating the inflammatory tissue context. Seven sections from seven independent donors were analyzed; donor was the biological and statistical unit. The primary within-donor comparison was evaluated using a two-sided exact paired sign-flip test with donor-level bootstrap estimation of the 95% confidence interval.

Within these epithelial regions, spots were classified as GART-undetected or GART-detected according to raw GART counts, and the external epithelial-program score was summarized separately for the two states within each donor. The program score was higher in GART-detected than in GART-undetected regions in all seven donors, with a mean within-donor difference of 0.0262 (95% CI 0.0140–0.0407; exact paired sign-flip P = 0.0156; **Figure 6**). Thus, the primary spatial analysis supported within-donor co-enrichment of the independently derived GART-associated epithelial program in regions where GART transcripts were detected.

Spatial maps of DNA-replication and DNA-repair programs (**Figure 6**), representative proliferation- and nucleotide-metabolism-associated markers including MYBL2, PCNA, PAICS, MCM7, GINS2, and OLFM4 (**Figure 6**), and MIF, CD74, and GART expression (**Figure 6**) further illustrated the regional heterogeneity and spatial coexistence of these features. These additional maps were interpreted descriptively and were not used to infer a causal MIF/CD74 niche, temporal repair process, or disease-specific signaling hierarchy.

### Human UC tissues show co-enrichment of GART with proliferative, ISC-like, and inflammatory-context features

In the GART/MIF/CD74 panel (HC, n = 18; UC, n = 25), UC tissues showed higher GART fluorescence intensity (location shift, 9.25; 95% CI, 6.44–11.57), MIF-high mucosal area (15.40 percentage points; 95% CI, 12.31–22.61), and CD74-positive cell density (mean difference, 879.74 objects/mm²; 95% CI, 755.96–1003.51; all P < 0.0001). CD74-positive cells within 50 μm of GART-positive regions also increased modestly (location shift, 10.97 percentage points; 95% CI, 0.32–27.16; P = 0.0467; **Figure 7A**).

**Figure 7:**
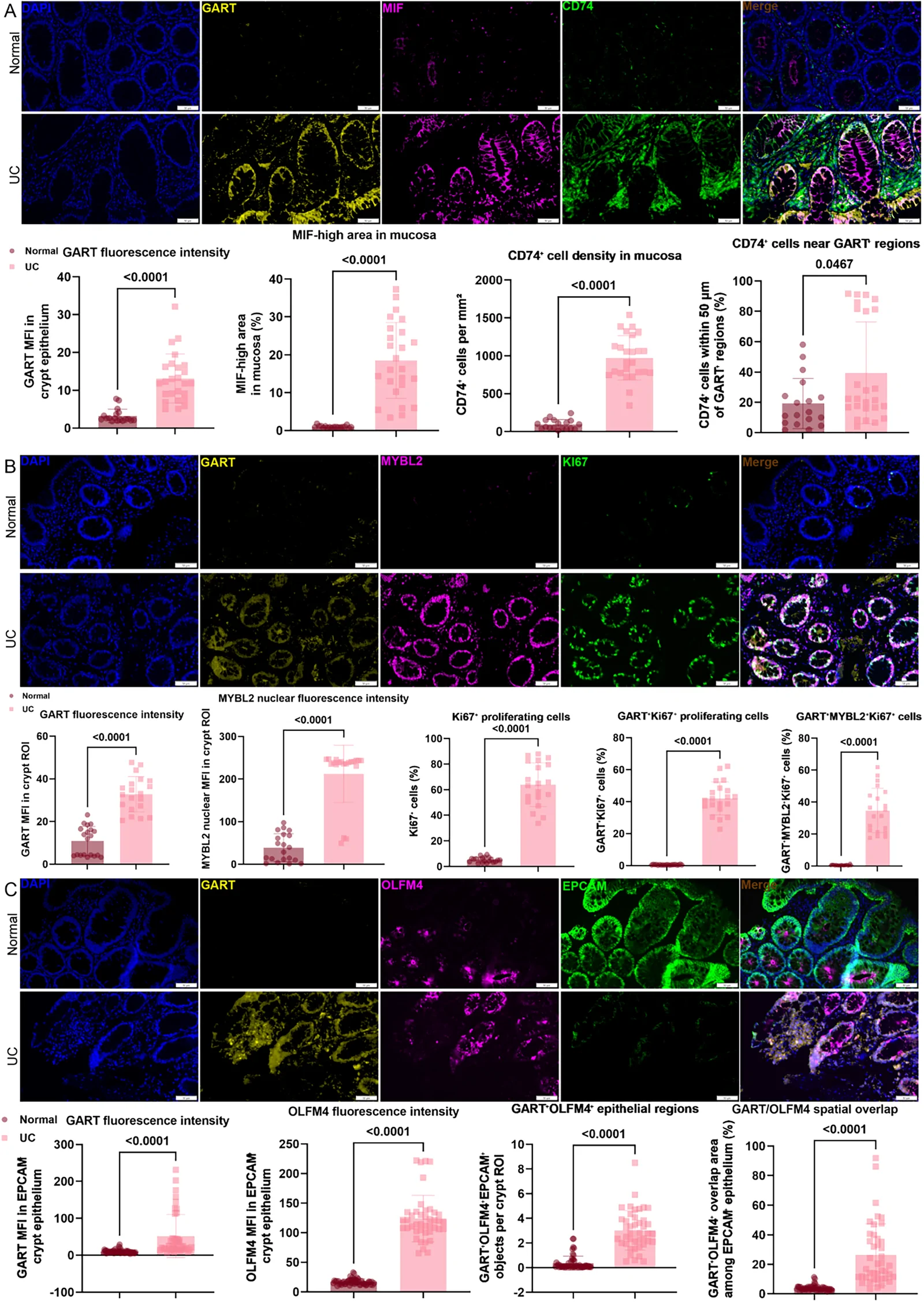
Human UC tissues show co-enrichment of GART with proliferative, ISC-like, and inflammatory-context features. **(A)** Representative immunofluorescence images of GART, MIF, and CD74 in healthy-control and UC colonic mucosa, with quantification of GART fluorescence intensity, MIF-high mucosal area, CD74-positive cell density, and CD74-positive cells near GART-positive regions. **(B)** Representative immunofluorescence images of GART, MYBL2, and Ki67, with quantification of GART fluorescence intensity, MYBL2 nuclear fluorescence intensity, Ki67-positive proliferating cells, GART/Ki67 double-positive cells, and GART/MYBL2/Ki67 triple-positive cells. **(C)** Representative immunofluorescence images of GART, OLFM4, and EPCAM, with quantification of GART fluorescence intensity, OLFM4 fluorescence intensity, GART/OLFM4 double-positive EPCAM-positive epithelial regions, and GART/OLFM4 spatial overlap in EPCAM-positive epithelium. DAPI was used for nuclear counterstaining. Scale bars are indicated in the images. P values are shown in the panels. Participant numbers were panel specific: **(A)** HC n=18 and UC n=25; **(B)** HC n=20 and UC n=20; **(C)** HC n=53 and UC n=42. Each plotted value represents one independent participant. Participant-level comparisons were two-sided and unpaired. Mann–Whitney U tests were used for all endpoints except CD74-positive cell density **(A)**, Ki67-positive cell percentage **(B)**, and GART/Ki67 double-positive cell percentage **(B)**, which were analyzed using Welch’s unpaired t tests.

In the GART/MYBL2/Ki67 panel (HC, n = 20; UC, n = 20), GART and MYBL2 fluorescence intensities were higher in UC (location shifts, 21.45 [95% CI, 17.12–27.49] and 194.06 [95% CI, 165.39–224.08], respectively; both P < 0.0001). Ki67-positive and GART/Ki67 double-positive cells increased by 58.67 (95% CI, 50.65–66.69) and 41.46 (95% CI, 36.63–46.29) percentage points, respectively, while GART/MYBL2/Ki67 triple-positive cells showed a location shift of 32.44 percentage points (95% CI, 23.19–41.74; all P < 0.0001; **Figure 7B**).

In the GART/EPCAM/OLFM4 panel (HC, n = 53; UC, n = 42), GART and OLFM4 fluorescence intensities were higher in UC (location shifts, 15.37 [95% CI, 10.49–25.01] and 99.42 [95% CI, 93.23–106.78], respectively). GART/OLFM4/EPCAM co-positive objects increased by 2.45 objects per crypt ROI, and GART/OLFM4 double-positive area increased by 14.04 percentage points (95% CI, 10.13–22.94; all P < 0.0001; **Figure 7C**). Together, these participant-level measurements support co-enrichment of GART with proliferative, ISC-like, and inflammatory-context features in UC.

### DSS-induced acute colitis is accompanied by systemic inflammation and epithelial barrier disruption

We next examined the GART-associated epithelial phenotype in a DSS-induced acute colitis model. DSS-treated mice showed progressive body-weight loss and increased disease activity index compared with control mice (**Figures 8A, B**). DSS treatment was also associated with an increased spleen index, reduced colon length, and marked histopathological injury characterized by epithelial and crypt damage and inflammatory-cell infiltration (**Figures 8C–E**), confirming the establishment of acute experimental colitis.

**Figure 8:**
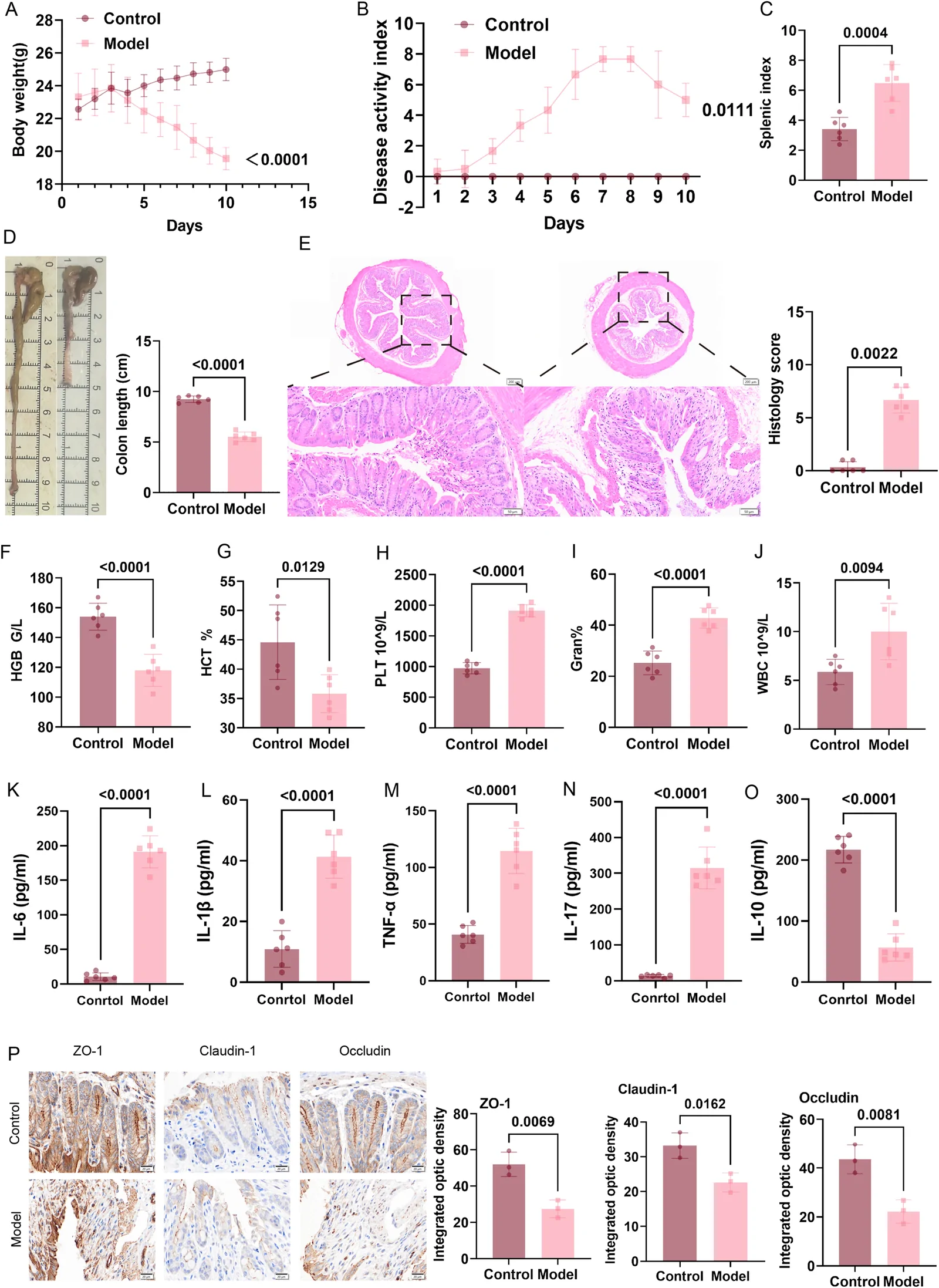
Establishment and characterization of the DSS-induced colitis mouse model. **(A)** Body weight changes during the experimental period. **(B)** Disease activity index. **(C)** Spleen index. **(D)** Representative colon images and colon length quantification. **(E)** Representative hematoxylin and eosin staining and histological score of colonic tissues. **(F–J)** Complete blood count parameters, including hemoglobin, hematocrit, platelet count, granulocyte percentage, and white blood cell count. **(K–O)** Serum levels of IL-6, IL-1β, TNF-α, IL-17A, and IL-10 measured by ELISA. **(P)** Representative immunohistochemical staining and quantitative analysis of epithelial barrier markers ZO-1, Claudin-1, and Occludin in colonic tissues. Longitudinal and systemic endpoints used n = 6 mice per group; immunohistochemistry used n = 3 mice per group. Each plotted point represents one mouse. Data are presented as mean ± SD. Two-group endpoints were analyzed using two-sided unpaired Student’s t-tests with equal variances. Benjamini–Hochberg adjustment was applied within the prespecified complete-blood-count, serum-cytokine, and barrier-protein endpoint families. Body weight and DAI were analyzed using mixed-effects models including time, group, and the group × time interaction. Three animals per group were randomly selected before staining and image quantification.

Complete blood count analysis showed lower hemoglobin and hematocrit levels and higher platelet counts, granulocyte percentages, and white blood cell counts in DSS-treated mice (**Figures 8F–J**). Serum ELISA further showed increased IL-6, IL-1β, TNF-α, and IL-17A concentrations and decreased IL-10 concentrations (**Figures 8K–O**). These systemic measurements were obtained from six mice per group. The differences across the related CBC and cytokine endpoints remained statistically significant after within-family Benjamini–Hochberg adjustment.

Immunohistochemical analysis of the imaging subset (n = 3 mice per group) showed lower expression of the epithelial barrier-associated proteins ZO-1, Claudin-1, and Occludin in DSS-treated colonic tissue (**Figure 8P**). These differences also remained significant after within-family multiple-testing adjustment and were consistent with epithelial barrier disruption in acute DSS colitis.

### GART-associated proliferative and ISC-like epithelial features in DSS-induced acute colitis

Multiplex immunofluorescence was then used to examine the tissue-level distribution of GART and associated epithelial markers in DSS colitis. In the GART/MYBL2/Ki67 panel, DSS-treated mice showed higher GART fluorescence intensity, MYBL2 nuclear fluorescence intensity, and a greater proportion of Ki67-positive cells. GART/Ki67 double-positive and GART/MYBL2/Ki67 triple-positive cells were also increased in the DSS group (**Figure 9A**), supporting co-enrichment of GART with a proliferative epithelial phenotype.

**Figure 9:**
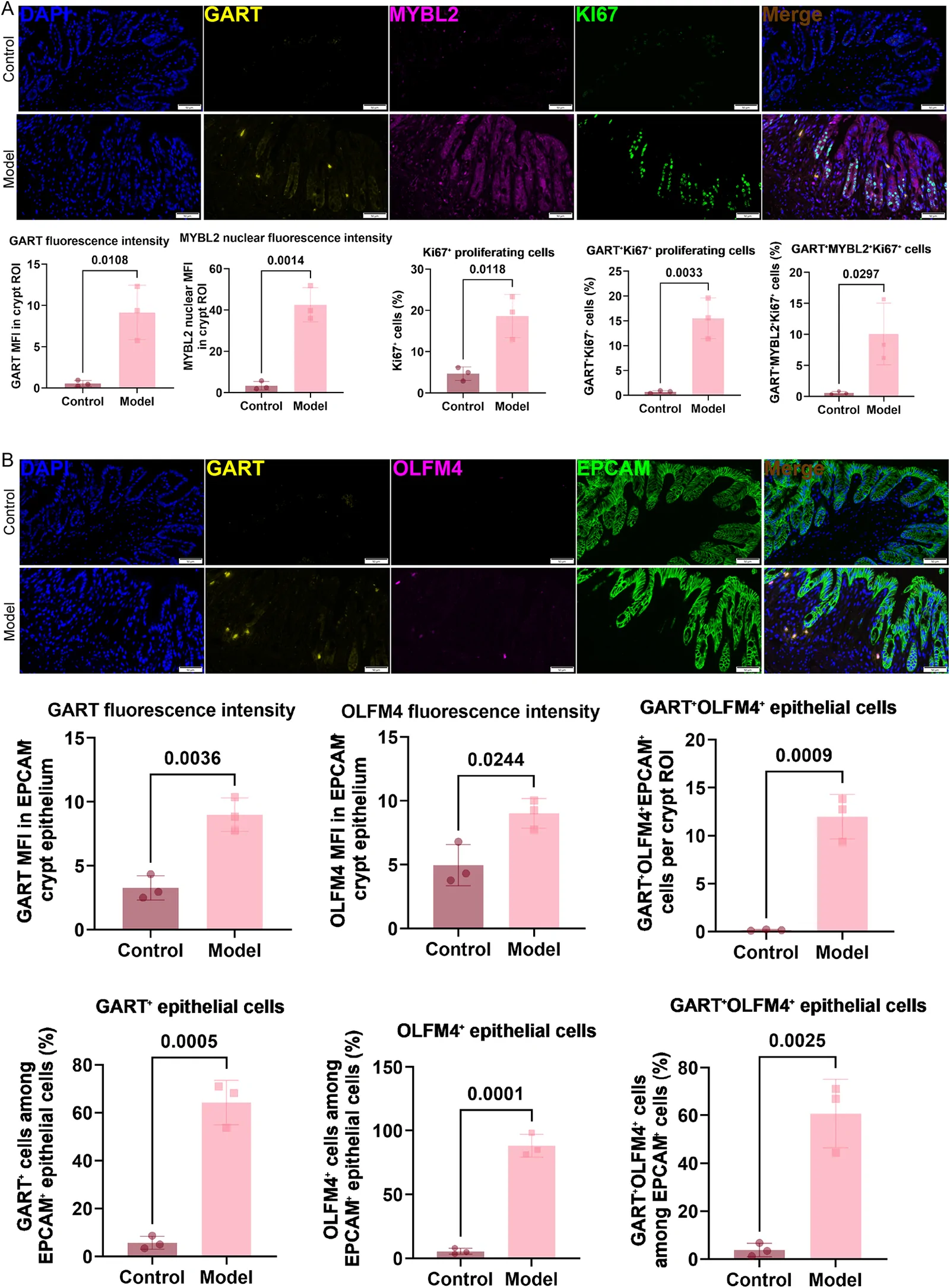
DSS colitis tissues show a GART-associated proliferative and ISC-like epithelial phenotype. **(A)** Representative immunofluorescence images of GART, MYBL2, and Ki67 in control and DSS model colonic tissues, with quantification of GART fluorescence intensity, MYBL2 nuclear fluorescence intensity, Ki67-positive proliferating cells, GART/Ki67 double-positive cells, and GART/MYBL2/Ki67 triple-positive cells. **(B)** Representative immunofluorescence images of GART, OLFM4, and EPCAM, with quantification of GART fluorescence intensity and OLFM4 fluorescence intensity in EPCAM-positive crypt epithelium, GART/OLFM4 double-positive epithelial cells, GART-positive epithelial cells, OLFM4-positive epithelial cells, and GART/OLFM4 double-positive cells among EPCAM-positive epithelial cells. DAPI was used for nuclear counterstaining. Scale bars are indicated in the images. Exact P values are shown in the panels. n = 3 mice per group; each plotted value represents one independent mouse. Data are presented as mean ± SD. Two-group comparisons were performed using two-sided unpaired Student’s t-tests with equal variances. P values were adjusted within this prespecified multiplex-immunofluorescence endpoint family using the Benjamini–Hochberg method. Three animals per group were randomly selected before staining and image quantification.

In the GART/EPCAM/OLFM4 panel, DSS-treated tissues showed higher GART and OLFM4 fluorescence intensities within EPCAM-positive crypt epithelium, together with increases in GART/OLFM4 co-positive epithelial cells and the proportions of GART-positive, OLFM4-positive, and GART/OLFM4 double-positive cells within the EPCAM-positive epithelial compartment (**Figure 9B**). These observations support an association between GART expression and an OLFM4-positive ISC-like epithelial phenotype in acute DSS injury.

Additional multiplex analyses showed higher GART fluorescence intensity, MIF-high area near GART-positive regions, CD74-positive cell density, and the proportion of CD74-positive cells near GART-positive regions in DSS-treated tissue (**Supplementary Figure S6A**). GART/PAICS spatial overlap, PAICS fluorescence intensity, and the proportion of PCNA-positive proliferating cells were also increased in the DSS group (**Supplementary Figure S6B****).** All multiplex imaging analyses used three mice per group, with the mouse as the biological unit. Together, these findings show co-enrichment of GART with proliferative, ISC-like, purine-metabolic, and inflammatory-context features during acute DSS-induced epithelial injury.

## Discussion

By integrating bulk and single-cell transcriptomics with spatial transcriptomics, human tissue multiplex immunofluorescence, and acute DSS mouse tissue analyses, this study identifies GART expression as a feature of a proliferative and purine-metabolic ISC-like epithelial state in UC. Several aspects of the revised analysis are important for interpreting this finding. Although bulk transcriptomic data showed an overall increase in GART expression in UC, the donor-aware single-cell analysis did not support a uniform disease-associated increase in GART detection across cohorts. Within UC-derived ISC-like epithelial cells, donor-paired pseudobulk analysis identified proliferation- and nucleotide-demand-associated transcriptional differences. Across the broader ISC-like epithelial atlas, covariate-adjusted module analyses further associated GART detection with purine-biosynthesis, DNA-replication, DNA-repair, and mitotic programs after accounting for donor structure, transcript complexity, and cell-cycle effects. Parallel analysis of other *de novo* purine-biosynthesis enzymes did not identify GART as uniquely altered. Thus, the principal finding is not a GART-specific regenerative mechanism, but rather the positioning of GART within a broader epithelial state characterized by increased proliferative activity and nucleotide demand.

This distinction is biologically relevant to the injured UC mucosa. Epithelial proliferation requires coordinated nucleotide production to sustain DNA synthesis, RNA synthesis, and cell-cycle progression, making *de novo* purine biosynthesis a plausible metabolic component of highly proliferative crypt epithelial states. The association of GART detection with PAICS, PCNA, MCM-family genes, GINS2, TYMS, and related replication programs is therefore consistent with increased nucleotide demand in proliferating ISC-like cells. Importantly, however, proliferation after injury should not automatically be equated with successful regeneration or mucosal healing. The GART-associated phenotype observed here may instead represent a metabolic adaptation of crypt epithelial cells responding to inflammatory injury. From a clinical perspective, this distinction may be useful because conventional assessment of UC primarily captures inflammatory burden and structural mucosal damage, whereas the metabolic state of the responding epithelium is less directly represented. A GART-associated multigene program may therefore warrant future evaluation as a tissue-level indicator of epithelial proliferative and nucleotide-demand states, although the present study does not establish its diagnostic, prognostic, or treatment-response value.

Prior functional studies provide context for these associations without establishing the same mechanism in our data. Intestinal nucleotide metabolism can be reprogrammed to support epithelial proliferation after disruption of homeostasis (29), while YAP/TAZ-dependent epithelial reprogramming and revival stem-cell states illustrate injury-associated plasticity rather than proving that the GART-detected cells identified here constitute those lineages (30, 31). OLFM4 can accumulate in inflamed intestinal epithelium under Notch and TNF-α signaling and has been linked to cell-protective responses, but OLFM4 expansion should not be equated with successful repair (32).

The revised pseudotime analysis further emphasizes the importance of separating proliferative state from temporal regeneration. GART-detected and GART-undetected ISC-like cells occupied different positions along the initial transcriptional continuum, but the association between GART expression and continuum position was markedly attenuated after exclusion of GART and predefined cell-cycle genes. The inferred trajectory therefore appears to capture, at least in substantial part, variation in proliferative and cell-cycle structure rather than a temporal transition from a GART-undetected to a GART-detected regenerative state. Similarly, scTenifoldKnk identified recurrent network associations involving nucleotide-metabolism, DNA-replication, and cell-cycle genes after simulated GART perturbation, but this represents perturbation of an inferred transcriptional network rather than experimental evidence of GART dependence. These analyses nevertheless provide a focused set of hypotheses for subsequent functional studies. In particular, direct GART perturbation in intestinal organoids, combined with measurements of nucleotide pools, DNA synthesis, proliferative capacity, and epithelial recovery after injury, will be necessary to determine whether GART merely marks increased nucleotide demand or contributes functionally to sustaining that state.

The microenvironmental and transcriptional analyses provide complementary perspectives on the GART-associated epithelial state. MYBL2 expression was higher in GART-detected ISC-like cells at the donor-paired level, consistent with its established role in cell-cycle progression, DNA replication, and proliferation (33). However, after adjustment for cell-cycle and technical covariates, regulon analysis did not support interpretation of MYBL2 as an independently activated GART-specific regulator. Likewise, HGNN/SigXTalk identified predicted receptor–transcription factor–target relationships involving MYBL2 and proliferation/nucleotide-metabolism-associated genes, but these relationships remain hypothesis-generating rather than evidence of a defined regulatory hierarchy.

At the microenvironmental level, donor-aware CellChat and LIANA+ analyses identified a consistent MIF/CD74-associated communication context around GART-detected ISC-like cells, while tissue imaging showed spatial coexistence of GART-positive epithelial regions with MIF- and CD74-associated inflammatory features. Prior functional work linking CD74 signaling to intestinal epithelial regeneration and mucosal healing supports the biological plausibility of this context (34), and MIF can signal through CD74 (35); however, our data show communication context and coexistence only and do not establish that MIF/CD74 regulates GART or is required for the GART-associated epithelial phenotype. Taken together, these findings suggest that an intrinsic proliferation/nucleotide-demand program and an inflammatory MIF/CD74-associated tissue context coexist with the GART-detected epithelial state, rather than supporting a linear MIF/CD74–MYBL2–GART signaling pathway.

Spatial and tissue-level analyses provided an important bridge between these computational observations and intact mucosal architecture. In seven independent spatial donors, an epithelial program derived independently from single-cell data was consistently higher in GART-detected than in GART-undetected epithelial regions. Human multiplex immunofluorescence further demonstrated co-enrichment of GART with OLFM4-positive ISC-like epithelial features, MYBL2/Ki67-associated proliferation, and MIF/CD74-associated inflammatory features across the corresponding panel-specific participant sets. Acute DSS colitis showed a similar tissue pattern, with increased GART accompanied by proliferative, OLFM4-associated ISC-like, PAICS/PCNA-associated purine-metabolic, and MIF/CD74-associated features in the context of marked inflammation and barrier disruption. These concordant tissue observations strengthen the biological relevance of the GART-associated state. Nevertheless, the DSS experiment represents acute epithelial injury with compensatory proliferation rather than a recovery-phase model; consequently, it supports an injury-associated proliferative response but cannot demonstrate successful epithelial repair or mucosal healing.

These findings have several potential translational implications, while also arguing against premature designation of GART as a therapeutic target. First, the cohort-dependent difference in GART detection indicates that GART alone is unlikely to function as a universal marker of UC activity. A broader epithelial program integrating GART with proliferation and nucleotide-metabolism features may prove more informative than a single-gene measurement and could ultimately complement inflammatory and histological indices when characterizing epithelial responses to treatment. Second, the association between proliferative ISC-like cells and *de novo* purine metabolism identifies nucleotide demand as a potentially important biological constraint during epithelial injury. This creates a testable translational hypothesis: metabolic states may help distinguish crypt regions that are actively responding to injury from relatively quiescent epithelium. However, because nucleotide synthesis is also required for normal epithelial proliferation, indiscriminate inhibition of GART or the purine-biosynthesis pathway could theoretically impair epithelial recovery rather than improve disease. Any therapeutic strategy directed at this pathway would therefore require direct functional studies defining when purine metabolism is adaptive, maladaptive, or dispensable. Third, combining epithelial metabolic markers with inflammatory-context markers such as MIF/CD74 may ultimately provide a more informative framework for studying heterogeneity in UC mucosal responses than either compartment alone.

The clinical relevance of this framework should therefore be tested prospectively rather than inferred from the present cross-sectional data. An important next step will be to determine whether the GART-associated epithelial program correlates with endoscopic and histological disease activity, subsequent mucosal improvement, treatment response, relapse, or progression toward dysplastic states. Longitudinal biopsies collected before and after effective therapy would be particularly informative because they could distinguish a transient injury-associated proliferative response from an epithelial state that persists despite inflammatory control. Such studies would also help determine whether increased purine-metabolic activity is beneficial during short-term epithelial restitution but potentially unfavorable when proliferation becomes prolonged or dysregulated. This distinction is clinically important because the same nucleotide-demand program that supports rapidly cycling epithelium after injury could have different consequences in chronic inflammation or neoplastic evolution.

Several limitations should be considered. Although the revised analyses used donor-aware pseudobulk and mixed-effects approaches for principal inference, individual-cell visualizations remain descriptive, and repeated biopsies or tissue fractions contributed to the underlying atlas. The number of healthy donors contributing sufficient ISC-like cells decreased under more stringent cell-number requirements, and several principal analyses were driven predominantly by SCP259 because GSE214695 did not satisfy the paired-cell criteria. GART detection based on raw transcript counts remains sensitive to transcriptional dropout and RNA complexity despite threshold sensitivity analyses and adjustment for nUMI, nGene, and cell-cycle covariates. Pseudotime and network perturbation remain computational and cannot establish temporal progression or functional dependence. CellChat, LIANA+, SCENIC, and HGNN/SigXTalk were derived from the same expression atlas and therefore represent complementary analyses rather than independent validation. The spatial cohort was small, and the human immunofluorescence panels involved different participant sets and incomplete clinical metadata. Detailed historical documentation of field/crypt selection, image-acquisition and quantitative-analysis blinding, and threshold-setting procedures for the archived human imaging analyses was unavailable, limiting retrospective assessment of potential image-selection and measurement bias. Multiplex staining demonstrated tissue-level co-localization but cannot establish ligand–receptor dependence or direct molecular interaction. Finally, acute DSS colitis does not reproduce chronic relapsing UC or demonstrate successful mucosal recovery. Direct perturbation experiments and prospective longitudinal human cohorts will therefore be required before the GART-associated state can be assigned causal or clinical utility.

In summary, the present study defines GART as a marker of a proliferative and purine-metabolic ISC-like epithelial state associated with inflammatory mucosal injury in UC. The convergence of donor-aware single-cell analyses, spatial co-enrichment, human tissue observations, and acute DSS tissue findings supports a relationship among epithelial nucleotide demand, proliferative activity, and the local inflammatory context, while comparison with other purine-biosynthesis enzymes argues against a uniquely GART-specific mechanism. This state-based framework provides a basis for future studies of epithelial metabolic adaptation in UC and for evaluating whether combined metabolic and inflammatory tissue features can inform clinically relevant measures of epithelial response, without implying that GART itself has yet been established as a therapeutic target or driver of mucosal repair.

## Conclusion

By integrating bulk transcriptomics, single-cell transcriptomics, spatial transcriptomics, human tissue multiplex immunofluorescence, and acute DSS mouse tissue analyses, this study identifies GART expression as a feature of a proliferative and purine-metabolic ISC-like epithelial state in UC. GART-detected ISC-like cells were associated with purine-biosynthesis, DNA-replication, DNA-repair, and proliferative programs, while spatial and tissue analyses showed co-enrichment with OLFM4-positive ISC-like, MYBL2/Ki67-positive proliferative, PAICS/PCNA-associated purine-metabolic, and MIF/CD74-associated inflammatory features. Together, these findings support an association between GART detection, proliferative nucleotide demand, and the inflammatory mucosal context, but do not establish GART as a specific causal driver of epithelial regeneration or repair.

## Data Availability

The datasets presented in this study can be found in online repositories. The names of the repository/repositories and accession number(s) can be found in the article/**Supplementary Material**.

## Supplementary material

Supplementary Table 1 Bulk transcriptomic cohort composition and meta-analysis. This table summarizes the retained healthy-control and non-neoplastic UC samples from GSE24287, GSE36807, GSE37283, and GSE87466, available clinical characteristics and exclusion criteria, cohort-specific GART effect sizes, and pooled random-effects meta-analysis statistics including heterogeneity measures.

Supplementary Table 2 Dataset-specific single-cell preprocessing, sample-to-donor mapping, GART-detection sensitivity, and analysis definitions. This table summarizes the source-study preprocessing procedures for GSE214695 and SCP259, the complete atlas-level sample-to-donor mapping, the subset of sample entries contributing to ISC-like analyses, primary and sensitivity GART-detection models, revision-specific integration and reclustering parameters, and complete gene-set definitions used for module scoring.

Supplementary Table 3 Donor-aware transcriptional and functional analyses of the GART-associated ISC-like epithelial state. This table summarizes donor-paired pseudobulk differential expression and gene-set enrichment analyses, functional-module mixed-effects models, cell-cycle-stratified analyses, and donor-aware comparisons with other de novo purine-biosynthesis enzymes.

Supplementary Table 4 Pseudotime sensitivity and five-run scTenifoldKnk virtual perturbation analyses. This table provides primary and feature-exclusion pseudotime sensitivity results, complete run-specific scTenifoldKnk perturbation rankings, recurrence metrics, consensus candidate genes, pathway-level summaries, and sampling-size sensitivity analyses.

Supplementary Table 5 Donor-aware communication, SCENIC, and HGNN/SigXTalk analyses. This table summarizes CellChat and LIANA+ donor-level communication analyses, covariate-adjusted SCENIC regulon results, and expression-supported HGNN/SigXTalk receptor–transcription factor–target candidate paths.

Supplementary Table 6 Spatial transcriptomic donor mapping and GART-associated epithelial-program analysis. This table provides the seven retained GSE189184 sections and donor mapping, the independently derived GART-associated epithelial program, epithelial ROI definition, donor-level spatial endpoints, and analysis parameters.

Supplementary Table 7 Participant characteristics of the human multiplex immunofluorescence cohorts. This table reports panel-specific participant characteristics for the GART/MIF/CD74, GART/MYBL2/Ki67, and GART/EPCAM/OLFM4 human tissue analyses. The participant was the independent biological and statistical unit.

Supplementary Table 8 Sequential TSA multiplex immunofluorescence protocol and technical information. This table provides antibody information, sequential staining order, polymer-HRP and TSA reagents, stripping conditions, optical filter specifications, acquisition metadata, and available technical QC information for the human and mouse multiplex panels.

Supplementary Table 9 Reagents used for mouse immunohistochemistry and serum ELISA assays. This table provides antibody information for ZO-1, Claudin-1, and Occludin immunohistochemistry and commercial ELISA kit information for the measured mouse serum cytokines.

Supplementary Figure s1 Random-effects meta-analysis of GART expression in UC. Forest plot showing cohort-specific Hedges’ g estimates and 95% confidence intervals for GSE24287, GSE36807, GSE37283, and GSE87466 and the pooled random-effects estimate. Cohort sample sizes were HC/UC = 25/27, 7/15, 5/4, and 21/87, respectively.

Supplementary Figure s2 Cohort-specific and donor-level analyses of GART detection in ISC-like epithelial cells. (A) Cohort-specific donor-aware estimates of the association between UC and GART detection. Donor, sample-entry, and cell numbers contributing to each cohort are indicated in the panel. (B) Donor-level GART-detected fractions in healthy and UC samples shown separately for GSE214695 and SCP259. Donor-aware cohort-specific models used 11 GSE214695 donors and 22 SCP259 donors; donor was the biological unit.

Supplementary Figure s3 Cell-cycle-stratified associations between GART detection and core functional programs. Heatmap showing covariate-adjusted associations between GART detection and purine-biosynthesis, DNA-replication, DNA-repair, and mitotic-cell-cycle module scores within G1, S, and G2/M phases. G1 included 30 donors, S included 25 donors, and G2/M included 21 donors; donor was the biological unit.

Supplementary Figure s4 Pseudotime sensitivity analysis after exclusion of GART and cell-cycle features. UMAP representation of the ISC-like epithelial transcriptional continuum after reconstruction with GART and predefined cell-cycle-associated genes excluded from the trajectory feature set. Cells are colored according to the recalculated pseudotime value. The reconstructed trajectory included 9,306 cells from 33 donors; cells are shown descriptively.

Supplementary Figure s5 Spatial distribution of GART and the external GART-associated epithelial program across all retained sections. (A) Spatial maps of GART expression across all seven retained GSE189184 sections, comprising two healthy-control and five UC sections, each representing an independent donor. (B) Spatial distribution of the independently derived single-cell-trained GART-associated epithelial program across the same seven sections. Donor was the biological unit. HC, healthy control; UC, ulcerative colitis.

Supplementary Figure s6 Additional inflammatory-context and purine-metabolic features in DSS colitis tissues. (A) Representative multiplex immunofluorescence images of GART, MIF, and CD74 in control and DSS-treated colonic tissues, with quantification of GART fluorescence intensity in crypt regions, MIF-high area within 50 μm of GART-positive regions, CD74-positive cell density in the mucosa, and the proportion of CD74-positive cells within 50 μm of GART-positive regions. (B) Representative multiplex immunofluorescence images of GART, PAICS, and PCNA, with quantification of GART/PAICS spatial overlap, PAICS fluorescence intensity in crypt regions, and the proportion of PCNA-positive proliferating cells. DAPI was used for nuclear counterstaining. n = 3 mice per group; each plotted point represents one independent mouse. Data are presented as mean ± SD. Two-group comparisons were performed using two-sided unpaired Student’s t-tests with equal variances. P values were adjusted within each prespecified multiplex-immunofluorescence panel using the Benjamini–Hochberg method. Three animals per group were randomly selected before staining and image quantification.

## Glossary

UC: Ulcerative colitis
ISC-like: Intestinal stem cell-like
scRNA-seq: Single-cell RNA sequencing
GEO: Gene Expression Omnibus
PCA: Principal component analysis
UMAP: Uniform manifold approximation and projection
TA: Transit-amplifying
GSEA: Gene set enrichment analysis
SCENIC: Single-cell regulatory network inference and clustering
AUCell: Area under the curve-based cell activity scoring
HGNN: Hierarchical graph neural network
DSS: Dextran sulfate sodium
DAI: Disease activity index
CBC: Complete blood count
HGB: Hemoglobin
HCT: Hematocrit
PLT: Platelet count
Gran%: Granulocyte percentage
WBC: White blood cell count
ELISA: Enzyme-linked immunosorbent assay
H&E: Hematoxylin and eosin
DAPI: 4′,6-diamidino-2-phenylindole
ROI: Region of interest
MFI: Mean fluorescence intensity
SD: Standard deviation
HRP: Horseradish peroxidase
DAB: 3,3′-diaminobenzidine
SMCs: Smooth muscle cells
T/NK: T cells/natural killer cells
IL-1β: Interleukin-1 beta
IL-6: Interleukin-6
IL-10: Interleukin-10
IL-17A: Interleukin-17A
TNF-α: Tumor necrosis factor-alpha
EPCAM: Epithelial cell adhesion molecule
OLFM4: Olfactomedin 4
MYBL2: MYB proto-oncogene like 2
Ki67: Marker of proliferation Ki-67
MIF: Macrophage migration inhibitory factor
CD74: CD74 molecule
PAICS: Phosphoribosylaminoimidazole carboxylase/phosphoribosylaminoimidazole succinocarboxamide synthetase
PCNA: Proliferating cell nuclear antigen
IMPDH2: Inosine monophosphate dehydrogenase 2
MCM6: Minichromosome maintenance complex component 6
MCM7: Minichromosome maintenance complex component 7
GINS2: GINS complex subunit 2
FEN1: Flap structure-specific endonuclease 1
TK1: Thymidine kinase 1
TYMS: Thymidylate synthase
CDK4: Cyclin-dependent kinase 4
ASCL2: Achaete-scute family BHLH transcription factor 2
RAN: RAN, member RAS oncogene family
MKI67: Marker of proliferation Ki-67
ZO-1: Zonula occludens-1
GART: Phosphoribosylglycinamide formyltransferase, phosphoribosylglycinamide synthetase and phosphoribosylaminoimidazole synthetase
HC: Healthy control
UMI: Unique molecular identifier
GLMM: Generalized linear mixed-effects model
LMM: Linear mixed-effects model
OR: Odds ratio
CI: Confidence interval
FDR: False discovery rate
BH: Benjamini–Hochberg
REML: Restricted maximum likelihood
TSA: Tyramide signal amplification
FFPE: Formalin-fixed paraffin-embedded
